# Targeting Cytoskeleton and Cell Motility: Past and Novel Strategies for Cancer Therapy

**DOI:** 10.32604/or.2026.082155

**Published:** 2026-08-13

**Authors:** Lucrezia Paradisi, Lorenza Trabalzini, Federica Finetti

**Affiliations:** Department of Biotechnology, Chemistry and Pharmacy, University of Siena, Siena, Italy

**Keywords:** Cancer therapy, metastasis, cytoskeleton, cancer cell motility, microtubule targeting agents, microfilament targeting agents, antimetastatic drugs

## Abstract

Cytoskeletal reorganization is fundamental to essential cellular processes, including shape maintenance, migration, adhesion cytokinesis, and phagocytosis, and its dysregulation is a hallmark of tumor progression. In cancer cells, altered cytoskeletal dynamics promote invasion and genomic instability resulting from mitotic defects. The actin and microtubule cytoskeletons are highly dynamic polymer networks that organize intracellular architecture, establish polarity, and generate the mechanical forces required for cell division and motility. Their dysregulation disrupts normal cell behavior and facilitates tumor invasion and metastasis. The cytoskeleton therefore represents a key source of potential therapeutic targets for inhibiting metastatic dissemination. This review focuses on recent anticancer strategies targeting cytoskeletal and motility-associated pathways, including microtubule-directed agents, actin-modulating compounds, inhibitors of focal adhesion signaling, modulators of intermediate filament dynamics, and regulators of cytoskeletal crosstalk. Collectively, these strategies underscore the central role of cytoskeleton in cancer progression and highlight its potential as a promising therapeutic target.

## 1 Introduction

The eukaryotic cytoskeleton is a highly dynamic network of biopolymers, comprising microtubules, actin filaments, and intermediate filaments that serves as the fundamental scaffold of cellular architecture [1,2,3]. Beyond providing structural support, it functions as a central integrator of mechanical and chemical signals regulating critical cellular processes, most notably cell growth and mitotic division [4].

In the context of oncology, the cytoskeleton undergoes extensive remodeling to meet the demands of uncontrolled proliferation. Microtubules, for instance, are essential for mitotic spindle formation, and their rapid assembly and disassembly dynamics are crucial for accurate chromosomal segregation. Consequently, the cytoskeleton has long been recognized as a major therapeutic target for inhibiting tumor proliferation, with microtubule-stabilizing and -destabilizing agents (e.g., taxanes and vinca alkaloids) representing cornerstones of conventional chemotherapy [5,6].

Emerging evidence has shifted the focus from mere tumor growth inhibition to the prevention of the lethal stages of cancer progression as metastatic cancer. Current studies increasingly target the actin cytoskeleton and its associated regulatory proteins, such as Rho GTPases, to block cancer metastasis. By disrupting the cytoskeletal rearrangements required for epithelial-mesenchymal transition (EMT), cell motility, and transendothelial migration, researchers aim to develop “migrastatics”, a new class of drugs designed to contain the primary tumor and prevent systemic dissemination [7,8].

The aim of this study is to provide a comprehensive analysis of pharmacological agents developed to date that specifically modulate cytoskeletal dynamics, thereby influencing the processes underlying cellular motility and metastasis.

## 2 Components and Regulators of the Cytoskeleton

The cytoskeleton is a dynamic network of highly organized and interconnected filaments that constitutes the fundamental structural framework of eukaryotic cells and, in a simpler form, of prokaryotic cells [9]. In eukaryotes, it comprises an intricate meshwork of protein filaments and associated motor proteins that regulate numerous essential cellular functions. These include the maintenance of cell shape, stabilization of organelle positioning, intracellular transport, and the provision of the mechanical support required for key processes such as cell division, cytokinesis, and motility [10].

The cytoskeleton is composed of three principal classes of protein filaments: microfilaments (MFs), microtubules (MTs), and intermediate filaments (IFs), which differ in diameter, with MTs being the largest and MFs the smallest [3,11]. In addition to these filament systems, three major superfamilies of cytoskeletal motor proteins, myosins, kinesins, and dyneins, play critical roles in force generation and intracellular transport. Myosins interact with actin filaments to mediate cortical contractions, vesicle trafficking, cytoplasmic streaming, and muscle contraction. Kinesin and dynein motors, which operate along microtubules, drive vesicle and organelle transport, power the beating of cilia and flagella, and participate in chromosome segregation during mitosis and meiosis as components of the mitotic and meiotic spindle apparatus [12,13].

### 2.1 Microfilaments

MFs are composed of actin polymers together with a diverse set of actin-binding proteins (ABPs). In cells, actin exists either as a monomeric globular form (G-actin) or as a polymeric filament (F-actin) [14]. Mammals possess six actin genes, each encoding a distinct protein isoform. Four isoforms, α-skeletal, α-cardiac, α-smooth, and γ-smooth actin, are predominantly expressed in skeletal, cardiac, and smooth muscle tissues. The remaining two isoforms, β-cytoplasmic and γ-cytoplasmic actin, are ubiquitously expressed. Despite their tissue-specific distribution, all six isoforms share highly conserved amino acid sequences, with no pair exhibiting less than 93% sequence identity [15].

MFs assemble through the polymerization of G-actin monomers into F-actin polymers. This process is highly dependent on the concentration of free actin monomers, which drives the equilibrium toward polymerization, and it also requires ATP and ABPs. The first step in filament formation is nucleation, during which three G-actin monomers assemble into a stable nucleus [16]. During elongation, ATP–G-actin monomers are added to the barbed (plus) end of the actin filament, promoting filament growth and reducing the local concentration of available G-actin. As G-actin levels decline, addition at the barbed end continues while subunits begin to dissociate from the pointed (minus) end. Elongation persists as long as the rate of monomer addition at the barbed end exceeds the rate of dissociation at the pointed end.

Because F-actin exhibits ATPase activity, ATP bound to newly incorporated monomers is hydrolyzed after polymerization, resulting in an ADP-rich filament. ATP-actin preferentially incorporates at the barbed end, whereas ADP-actin dissociates more readily from the pointed end, giving rise to treadmilling dynamics [14,17]. Profilin is an actin-binding protein that regulates actin polymerization by binding monomeric actin and masking one of its actin–actin interaction interfaces. In doing so, profilin sequesters actin from the pool of polymerization-competent monomers, thereby inhibiting spontaneous filament assembly [17].

MFs are essential for a wide range of cellular functions. One of their primary role is providing mechanical stability; for example, microvilli on the surface of intestinal and renal epithelial cells are supported by bundled actin filaments [18]. MFs also drive changes in cell shape, such as during cytokinesis, when an actomyosin contractile ring constricts the dividing cell to separate daughter cells [19]. Furthermore, actin, together with myosin motors, generates contractile force in processes such striated muscle contraction [20]. MFs also support diverse forms of cell motility, ranging from whole-cell migration to intracellular transport of organelles [21,22,23]. While myosin motors generate much of the mechanical force required for these processes, actin filaments can also generate force independently through actin polymerization, particularly at lamellipodia at the leading edge of migrating cells [24] and during ameboid movement [25,26].

### 2.2 Microtubules

MTs are highly dynamic structures that play key roles in determining cell shape and supporting numerous forms of cellular movement, including cell locomotion, intracellular transport of organelles, and chromosome segregation during mitosis. They are composed of α/β-tubulin heterodimers that assemble into long polymers. A third tubulin isoform, γ-tubulin, is specifically concentrated at the centrosome, where it is essential for initiating microtubule assembly [27].

Structurally, microtubules are hollow tubes; this architecture makes them more rigid than other cytoskeletal filaments and enables them to span the entire eukaryotic cell. Each microtubule consists of linear strands known as protofilaments. Most microtubules are composed of 13 protofilaments, each formed by the head-to-tail alignment of α/β-tubulin heterodimers [28]. When the concentration of tubulin dimers exceeds a critical threshold, polymerization is initiated.

Microtubules also exhibit intrinsic structural polarity that is essential for their biological functions. Within each protofilament, α-tubulin subunits are exposed at one end and the β-tubulin subunits at the opposite end; these are referred to as the minus and plus ends, respectively [29]. This polarity underlies the different rates of tubulin addition and loss at the microtubule ends: the plus end grows more rapidly, whereas the minus end grows more slowly [28].

MTs are inherently dynamic, undergoing continuous cycles of growth, shrinkage, and regrowth. This energy-dependent behavior, known as dynamic instability, is crucial for numerous cellular functions, including mitosis [30,31], interactions with actin filaments [32,33], generation of pushing and pulling forces [34], and cytoskeleton remodeling [35,36].

During polymerization, both α- and β-tubulin subunits within each dimer bind GTP. The GTP associated with α-tubulin is non-exchangeable and serves a structural role, whereas the GTP bound to β-tubulin is rapidly hydrolyzed to GDP shortly after incorporation of new dimers. Because GDP-tubulin has different kinetic properties and is more prone to depolymerization, its presence at the microtubule tip destabilizes the polymer. Although GDP-tubulin within the lattice cannot spontaneously dissociate, GDP-tubulin exposed at the microtubule end readily dissociates. Since tubulin dimers are added to the microtubule only in the GTP-bound state, a stabilizing “GTP cap” forms at the growing tip and protects the polymer from disassembly. When GTP hydrolysis reaches the tip, the microtubule undergoes rapid depolymerization and shrinkage. The switch between growth and shrinkage is known as “catastrophe”. Growth can resume when GTP-tubulin is incorporated again at the tip, re-establishing the protective cap; this process is referred to as “rescue” [30,37].

The role of MTs in cell division makes them attractive targets for anticancer drugs with antiproliferative activity.

### 2.3 Intermediate Filaments

IFs span the cytoplasm, linking the nuclear envelope to the plasma membrane as well as to actin filaments and microtubules. They play a key role in determining cell morphology and mechanical properties [38]. IFs derive their name from their 10-nm diameter, which is smaller than that of microtubules (24 nm) but larger than that of microfilaments (7 nm).

Unlike microfilaments and microtubules, which are built from a limited number of widely expressed actin or tubulin isoforms, intermediate filaments show marked cell-type-specific variation in protein composition [39]. More than 50 IF proteins have been identified and grouped into six classes based on similarities in their amino acid sequences. These include keratins in epithelial cells [40]; vimentin in fibroblasts, smooth muscle cells, and white blood cells [41]; desmin in muscle cells [42]; neurofilaments in mature neurons, where they are especially abundant in motor neuron axons [43]; nuclear lamins in most eukaryotic cells, where they act as structural components of the nuclear envelope and form a characteristic meshwork beneath the nuclear membrane [44]; and nestin, expressed in many cell types during development and largely absent in adult tissues [45].

All IF proteins are characterized by a central α-helical rod domain, which is divided into three segments of conserved length containing distinctive hydrophobic sequences. This rod domain is flanked by N-terminal head and C-terminal tail domains. The N- and C-terminal regions of the rod domain are highly conserved across all IF proteins, and point mutations within these regions can markedly disrupt IF organization and dynamics [46,47].

IFs were long considered a largely static network that maintains overall cell shape and architecture, in contrast to actin filaments and microtubules, which undergo rapid cycles of polymerization and depolymerization. However, live-cell studies have shown that IFs are, in fact, highly dynamic structures capable of shortening, elongating, and reorganizing [48]. Mutations that disrupt IF dynamics often impair filament assembly and promote the formation of protein aggregates [49].

Phosphorylation plays a central role in regulating IF behavior: it drives keratin depolymerization and is a key modulator of vimentin dynamics and neurofilament organization [50]. Beyond classical end-dependent polymerization, IFs can undergo end-to-end annealing and subunit exchange along the length of existing filaments, as demonstrated for vimentin and neurofilaments [51].

IF dynamics are further coordinated with those of microtubules and actin filaments. Subunit exchange relies on microtubules, and motor-driven transport by kinesins and dynein moves both short and long IF fragments throughout the cytoplasm [52,53]. Interactions with actin filaments additionally contribute to IF remodeling through actin retrograde flow [54].

Although IF proteins are increasingly recognized as contributors to tumor progression, their roles in cancer cell migration, invasion, and metastasis remain poorly defined. In epithelial tissues, IF keratins provide essential structural connections by anchoring at cell–cell junctions, which are often disrupted during metastatic dissemination. Nestin is overexpressed in multiple tumor types, and its expression correlates with aggressive growth, enhanced metastatic capacity, and poor clinical outcomes in cancers such as pancreatic and prostate carcinoma, melanoma, and glioblastoma [39]. Vimentin is also upregulated in various malignancies and is associated with increased invasiveness and an unfavorable prognosis. Despite these observations, the mechanistic contributions of nestin and vimentin to tumor biology are still not fully understood [27].

## 3 Antiproliferative Compounds: Microtubule-Targeting Agents

Microtubule-targeting agents (MTAs) are compounds with antimitotic properties and are among the most successful classes of classical chemotherapeutics. By binding tubulin, these spindle poisons disrupt microtubule dynamics and prevent the formation of a functional bipolar spindle. As a result, chromosomes in dividing cells fail to establish proper attachments to spindle microtubules, leading to chronic activation of the spindle assembly checkpoint and a prolonged mitotic arrest. Cells trapped in mitosis ultimately undergo apoptosis [55]. Mitotic arrest is an effective strategy for inhibiting tumor cell proliferation.

Most MTAs are natural products or semi-synthetic derivatives, originating from a wide range of terrestrial and marine sources, including plants, microorganisms, and sponges. Based on their primary effects on microtubule behavior, MTAs are generally classified as either microtubule-stabilizing or microtubule-destabilizing agents [6]. Microtubule-stabilizing agents (MSAs) exert their effects by promoting microtubule stabilization and inhibiting depolymerization, ultimately leading to cell death. The most well-known representatives of this group currently used in anticancer therapy are the taxanes and epothilones [56]. Microtubule-destabilizing agents (MDAs) act by inhibiting microtubule polymerization, resulting in microtubule shortening. Notable examples include the vinca alkaloids and colchicine-binding site (CBS) inhibitors, such as combretastatins [57] (Table 1).

Despite their efficacy as anticancer agents, MTAs are limited by several drawbacks, including systemic, hematologic, and neurotoxicity, the rapid emergence of multidrug and tubulin-based resistance, poor blood–brain barrier penetration [58], and suboptimal pharmacokinetics [59]. A major limitation of currently available MTAs is their toxicity, which often necessitates dose reduction or even treatment discontinuation.

Peripheral neuropathy is among the most common and clinically significant side effects, arising from damage to microtubules in the long axons of peripheral nerves. This can lead to symptoms that are only partially reversible or, in some cases, permanent [60].

Resistance to MTAs is a multifactorial phenomenon involving several molecular mechanisms. One key contributor is the overexpression of P-glycoprotein (P-gp), an ATP-binding cassette transporter that actively exports drugs from cancer cells, thereby promoting a multidrug resistance (MDR) phenotype [61,62,63]. In addition, dysregulation of apoptosis-related proteins, particularly members of the Bcl-2 family, and alterations in drug–target interactions, contribute to resistance [64]. Changes in β-tubulin isotype expression and mutations, including overexpression of class III β-tubulin, have also been implicated in the development of resistance *in vitro* [59].

Interestingly, in 2012, Mitchison and colleagues introduced the concept of the “proliferation rate paradox,” which fundamentally challenged the long-held belief that MTAs—such as taxanes and vinca alkaloids—work solely by freezing cancer cells in mitosis. If cancer cells divide slowly, sometimes taking weeks between divisions, yet a brief course of chemotherapy successfully shrinks a tumor, the math does not fully support a “mitosis-only” mechanism [65].

The hypothesis that MTAs trigger a chain reaction provides a compelling way to resolve this paradox. Instead of needing to kill every cell directly, these drugs may eliminate a small fraction of actively dividing cells, which then “signal” the destruction of their neighbors. Dying mitotic cells can release Damage-Associated Molecular Patterns (DAMPs), recruiting the immune system to the tumor site and potentially leading to the clearance of nearby non-dividing (interphase) cancer cells [58,66,67].

Furthermore, since cells spend the vast majority of their life in interphase, research has shifted toward how MTAs disrupt the cellular “highways” during non-dividing phases. In interphase, microtubules are responsible for intracellular transport, moving organelles, mRNA, and proteins. MTAs can “clog” these tracks, preventing essential proteins from reaching their destinations. Microtubules also regulate nuclear trafficking; since many transcription factors and DNA-repair proteins rely on microtubules to enter the nucleus, MTAs can impair the cell’s ability to repair itself or respond to growth signals.

In addition, some MTAs may act as vascular disrupting agents (VDAs). By collapsing the microtubule cytoskeleton of endothelial cells lining tumor blood vessels, they shut down blood flow, causing massive necrosis of cancer cells regardless of their division state [68].

Moreover, current evidence suggests that MTAs often induce mitotic slippage: instead of dying immediately in mitosis, cells “leak” back into interphase without proper division. These cells become tetraploid, multinucleated cells and are then faced with different fates: arrest in the G1 phase of the cell cycle, post-slippage cell death, or continued cycling as genomically unstable cells. Mitotic slippage may influence treatment outcomes and confer acquired resistance following antimicrotubule drug treatment [69].

### 3.1 Microtubule-Stabilizing Agents

Taxanes are potent chemotherapeutic agents of natural or semi-synthetic origin, structurally related and derived from the yew tree (genus *Taxus*). Paclitaxel, docetaxel, and cabazitaxel are key drugs in the treatment of various cancers, including ovarian, breast, prostate, and lung cancers [70]. First-generation taxanes, such as paclitaxel and docetaxel, have significantly improved the overall survival in cancer patients. However, intrinsic and acquired resistance remain major limitations to their clinical efficacy.

Cabazitaxel, a newer-generation taxane, was developed to overcome resistance, as it has a lower affinity for P-gp. In fact, the FDA approved cabazitaxel in combination with prednisone in 2010 for the treatment of patients with metastatic hormone-refractory prostate cancer who had previously received a docetaxel-prednisone regimen [71].

Taxanes bind to β-tubulin within a luminal pocket of microtubules through a combination of polar and hydrophobic interactions, resulting in suppression of microtubule dynamics. This stabilization interferes with proper mitotic progression, slowing or preventing the transition from metaphase to anaphase. Cells affected by this blockade may undergo apoptosis or exit mitosis aberrantly [71]. In addition to their effects on mitosis, taxanes disrupt microtubule-dependent processes, leading to mitosis-independent effects such as impaired intracellular trafficking. They also exhibit antiangiogenic properties, mediated through inhibition of endothelial cell proliferation and migration [72].

Epothilones represent a more recently developed class of anticancer agents compared with taxanes. Epothilone A and epothilone B are secondary metabolites originally isolated from the myxobacterium *Sorangium cellulosum*. Ixabepilone, an epothilone B analog, was approved by the FDA in 2007 for the treatment of locally advanced or metastatic breast cancer, either as monotherapy or in combination with capecitabine [73].

Other epothilones that have been developed and are under investigation in clinical trials include patupilone (phase I-III), sagopilone (phase I and II), KOS-1584 (phase II) [74], and utidelone (phase I-III) [75]. Epothilones also bind to β-tubulin, leading to suppression of microtubule dynamics, arrest of the cell cycle at the G_2_/M phase, and subsequent induction of apoptosis.

Although taxanes and epothilones share the same binding site on β-tubulin, epothilones retain growth-inhibitory activity in cells that are resistant to taxanes due to P-gp overexpression or β-tubulin mutations. These findings suggest that epothilones interact with tubulin in a manner distinct from taxanes and that their efficacy is not significantly affected by P-gp–mediated drug efflux. This is clinically relevant, as epothilones may represent an effective treatment option for taxane-resistant cancers [73].

### 3.2 Microtubule-Destabilizing Agents

Colchicine was the first MDA to be discovered. Although it potently disrupts mitotic progression, its clinical use is limited by high toxicity and a narrow therapeutic index [55].

Combretastatins bind to the colchicine binding site on the β-tubulin subunit. Among them, combretastatin A4 (CA-4) is the most active compound, exhibiting strong antiproliferative and antiangiogenic properties. CA-4 shows potent cytotoxic activity against a wide range of human cancer cell lines, including MDR cells; however, its clinical application is hampered by poor bioavailability due to low water solubility, a short biological half-life, and *cis-trans* isomerization [76]. To overcome these limitations, phosphate prodrugs of combretastatin A4 have been developed. Combretastatin A4 phosphate (CA-4P) is currently under investigation as a treatment for anaplastic thyroid cancer, either as monotherapy or in combination with carboplatin and paclitaxel (NCT00060242, NCT00507429). In solid tumors, CA-4P has been shown to predominantly exert antiproliferative effects within the tumor core, often leaving a rim of viable tumor cells at the periphery. Therefore, combination strategies incorporating CA-4P with other therapeutic modalities are preferred to overcome this limitation and enhance overall antitumor efficacy [77].

Eribulin is a synthetic analogue of the marine sponge natural product halichondrin B [78] that binds to the plus ends of microtubules via the vinca domain on the polymerization surface of β-tubulin. It selectively inhibits microtubule polymerization and elongation without affecting shortening, leading to the formation of nonproductive tubulin aggregates, G_2_/M cell-cycle arrest, and apoptosis following prolonged mitotic blockade. This mechanism is distinct from that of other microtubule-targeting agents, such as taxanes, epothilones, and vinca alkaloids, which affect both microtubule growth and shortening. Owing to their high proliferative rate, cancer cells are particularly susceptible to this mode of action [79].

In addition to its antimitotic effects, eribulin modulates the tumor microenvironment by promoting reversal of EMT, potentially through inhibition of TGF-β/Smad signaling, and by reducing cancer stem cell populations. Eribulin has also been shown to induce vascular remodeling and attenuate immunosuppressive tumor features [80]. It has demonstrated significant antitumor activity in multiple preclinical models, including colorectal, non–small-cell lung, melanoma, glioblastoma, and ovarian cancers [80]. The FDA approved eribulin in 2010 for the treatment of patients with metastatic breast cancer who had previously received an anthracycline and a taxane, and in 2015 for patients with unresectable or metastatic liposarcoma who had received a prior anthracycline-containing regimen.

Maytansine is an ansa macrolide initially isolated from the plant *Maytenus ovatus* that exerts its antimitotic activity by binding to tubulin and inhibiting microtubule assembly. The maytansine-binding site is located on an exposed region of the β-tubulin subunit, adjacent to the vinca alkaloid–binding site, at the interface between two tubulin heterodimers. Binding to this site induces microtubule destabilization through concentration-dependent mechanisms: at low concentrations, maytansine inhibits the addition of tubulin heterodimers to the plus ends of growing microtubules, whereas at higher concentrations it sequesters tubulin into nonpolymerizable tubulin–drug complexes [6]. Maytansine has demonstrated potent cytotoxic activity against a variety of cancer cell lines and effectively inhibits tumor growth and proliferation *in vivo* [81]. However, its clinical application has been limited by a narrow therapeutic window, as human clinical trials revealed significant toxic side effects, particularly affecting the gastrointestinal tract and nervous system. To overcome these limitations, antibody–drug conjugates (ADCs) have been developed to enhance the therapeutic potential of maytansine while reducing systemic toxicity through selective targeting of cancer cells. In 2012, the FDA approved ado-trastuzumab emtansine, an ADC composed of the anti-HER2 monoclonal antibody trastuzumab linked to the microtubule inhibitor emtansine (DM1), a synthetic derivative of maytansine. This ADC is indicated as monotherapy for the treatment of patients with HER2-positive metastatic breast cancer who have previously received trastuzumab and a taxane, either sequentially or in combination.

Auristatins are highly potent synthetic antimitotic agents derived from dolastatin 10. They act as microtubule-destabilizing agents by binding to tubulin at or near the vinca alkaloid–binding site. A prominent synthetic derivative is monomethyl auristatin E (MMAE). The mechanisms by which these molecules act on tubulin dimers include induction of curved aggregates and inhibition of nucleotide exchange [82]. This agent is used as the cytotoxic payload in several FDA-approved ADCs, including brentuximab vedotin for Hodgkin lymphoma and systemic anaplastic large cell lymphoma (sALCL); enfortumab vedotin for locally advanced or metastatic urothelial carcinoma; telisotuzumab vedotin for locally advanced or metastatic non-squamous non–small-cell lung cancer (NSCLC) with high c-Met protein overexpression; polatuzumab vedotin-piiq for relapsed or refractory diffuse large B-cell lymphoma; and tisotumab vedotin-tftv for recurrent or metastatic cervical cancer (Table 1).

**Table 1:** Microtubule-targeting compounds with antiproliferative properties.

Drugs	Class	Target	Mechanism of Action	Mechanism of Resistance	Evidences	Drug Development	References
**Paclitaxel**	Taxanes	Microtubules	Microtubules stabilization Antimitotic Impaired intracellular traffic Antinagiogenic	Pgp mediated efflux Overxpression of genes (BCRP, TRAG-3, EDIL-3) Cytocrome P450 metabolism p53 mutation	*in vitro* *in vivo* clinical	Clinically approved	[72,83]
**Docetaxel**				Pgp mediated efflux Tubulin alterations (βIII-tubulin) Androgen Receptor signalling ERG rearrangments PI3K/Akt activation			[72,84]
**Cabazitaxel**				Reduced substrate affinity for Pgp Higher expression of βIII-tubulin Decreased BRCA1 expression Induction of EMT			[72,85]
**Ixabepilone**	Epothilones		Microtubules stabilization Antimitotic	Reduced susceptibility to Pgp efflux Retain efficacy in βIII-tubulin overexpressing tumors	*in vitro* *in vivo* clinical	Clinically approved	[86]
**Patupilone**						Clinical trial	
**Sagopilone**				Reduced substrate affinity for Pgp–mediated drug efflux			
**KOS-1584**				Studied for activity in taxane-resistant disease Reduced substrate affinity for Pgp–mediated drug efflux			
**Utidelone**				Reduced substrate affinity for Pgp–mediated drug efflux			
**Combretastatin A4 phosphate**	Combretastatins		Microtubules destabilization Antiproliferative Antiangiogenic	Not affected by Pgp overexpression Tumour vascular adaptations	*in vitro* *in vivo* clinical	Clinical trial	[87]
**Eribulin**	Synthetic analogue of halichondrin B		Microtubules destabilization: inhibition of microtubule growth Antiproliferative	Pgp and ABCC11-mediated efflux Altered microtubule response following drug treatment (acetylation changes) Activation of survival pathways (PI3K/AKT, NF-κB) Reduced capacity for endothelial transdifferentiation and increased PD-L1 expression	*in vitro* *in vivo*	Clinically approved	[88,89]
**Ado-trastuzumab emtansine**	ADC (maytansinoid-based)		Targeted microtubule destabilization in a concentration-dependent manner	Reduced HER2 expression Defects in intracellular trafficking and lysosomal degradation Efflux pumps (Pgp overexpression) and transporters (SLC46A3) limiting intracellular accumulation Adaptation to microtubule damage Activation of pro-survival pathways (PI3K–AKT–mTOR, YES1) Impaired mitotic response (cyclin B1 dysregulation)	*in vitro* *in vivo* clinical	Clinically approved	[90,91,92]
**Brentuximab vedotin**	ADC (auristatin-based)		Targeted microtubule destabilization	Efflux pumps Pgp–mediated drug export Reduced sensitivity to MMAE Target antigen alterations (CD30 or Nectin-4 downregulation)	*in vitro* *in vivo* clinical	Clinically approved	[93,94,95]
**Enfortumab vedotin**							
**Telisotuzumab vedotin**					*in vitro* *in vivo*		
**Polatuzumab vedotin-piiq**							
**Tisotumab vedotin-tftv**							

## 4 Cancer Metastasis

**Figure 1:**
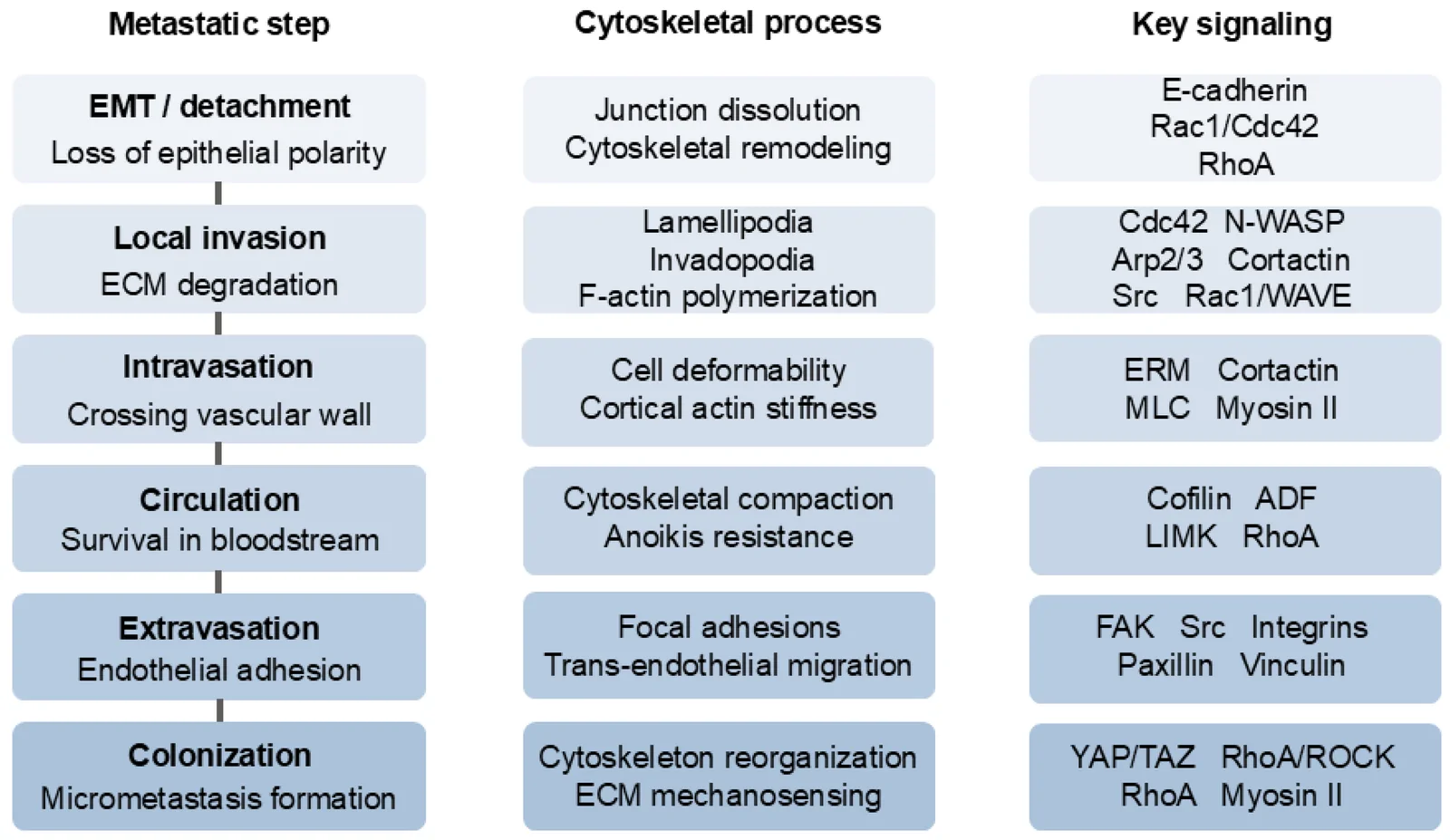
Schematic representation of the metastatic cascade. Cancer cells detach from the primary tumor through EMT, accompanied by E-cadherin downregulation and cytoskeletal remodeling. Cells subsequently degrade the extracellular matrix via invadopodia formation, intravasate into the vasculature, and survive in the bloodstream through cytoskeletal compaction and resistance to anoikis. Following extravasation across the endothelial barrier, cancer cells colonize distant organs via ECM mechanosensing and cytoskeletal reorganization. Each step is coupled with specific intracellular signaling cascades, including Rho GTPases (RhoA, Rac1, Cdc42), actin-regulatory proteins (Arp2/3, N-WASP, cofilin), and focal adhesion components (FAK, Src, integrins).

Metastasis remains the most significant obstacle in effective cancer treatment and is responsible for the majority of cancer-related mortality [96]. It arises from the dissemination of malignant cells from the primary tumor to distant organs. This dissemination is a highly regulated, multistep process commonly described as the metastatic cascade. To establish secondary lesions, tumor cells must traverse a sequence of distinct steps, including detachment from the primary tumor, migration and invasion of surrounding tissue, intravasation into the vasculature, survival during transit through resistance to anoikis, extravasation into distant tissues, and ultimately, colonization and outgrowth at the secondary site (Fig. 1).

The successful completion of these stages requires extensive spatial and temporal reorganization of the cytoskeleton, which underlies the ability of cancer cells to alter their shape and migrate [27]. Although cell migration is crucial for physiological processes such as development, immune surveillance, and tissue repair, its dysregulation is involved in cancer invasion and dissemination [97].

Tumor cell movement relies on four coordinated steps: protrusion, adhesion, contraction, and retraction. In response to extracellular gradients of growth factors or chemokines, cells polarize and generate actin-driven membrane protrusions towards the cue [98]. These protrusions, such as lamellipodia, filopodia, pseudopodia, invadopodia, or podosomes, are stabilized by linkage of the actin cytoskeleton to the extracellular matrix (ECM), while actomyosin contraction generates traction forces. Contraction also promotes adhesion turnover at the rear of the cell, enabling retraction and forward translocation [99,100].

ECM components interact with a variety of transmembrane receptors, including integrin and non-integrin receptors such as CD44, discoidin domain receptors, CD26, immunoglobulin superfamily receptors, and surface proteoglycans. Engagement of integrins and other adhesion receptors recruits surface proteases, such as matrix metalloproteinases (MMPs) and cathepsins, to attachment sites, which in turn degrade ECM components near the cell surface [101,102].

Actin filaments locally elongate and assemble through the action of cross-linking proteins such as α-actinin, myosin II, and others. Branched actin networks beneath the inner leaflet of the plasma membrane are referred to as cortical actin, whereas cytoplasmic bundles and elongated actin filament cables are known as stress fibers [103]. Myosin II, the major motor protein in non-muscle eukaryotic cells, mediates actin filament contraction. Assembly and contraction of stress fibers are primarily regulated by the small GTPase Rho and its downstream effector, Rho-associated protein kinase (ROCK). In contrast, the cortical actin network is regulated by myosin light chain kinase (MLCK) [104,105].

Cancer cells can spread as individual cells, a process known as ‘individual cell migration’, or as multicellular structures such as solid strands, sheets, files, or clusters, known as ‘collective migration’. In many tumors, both individual and collective modes coexist. Whereas leukemias, lymphomas, and most solid stromal tumors such as sarcomas disseminate primarily as single cells, epithelial tumors often employ collective migration mechanisms [106].

Individual cancer cells can disseminate through mesenchymal or amoeboid modes of migration. Amoeboid migration is characterized by rapid movement and a rounded yet highly deformable morphology. It occurs without strong adhesion or significant proteolytic matrix degradation and is characterized by weak interactions with the ECM [107]. The high deformability of tumor cells, driven by dynamic remodeling of the cortical actin cytoskeleton, enables them to squeeze through narrow ECM gaps [25,26,108]. The nucleus also undergoes substantial compression to facilitate passage [109,110]. Movement is further supported by bleb-like membrane protrusions that interact with and sense the surrounding environment through mechanotransduction [26]. These blebs, along with cortical actin dynamics, are primarily regulated by RhoA–ROCK signaling [107].

Mesenchymal migration is a characteristic mode of movement for fibroblasts, endothelial cells, and smooth muscle cells [111]. In tumors, it is commonly observed in connective tissue- or bone marrow-derived malignancies, as well as in certain poorly differentiated epithelial cancers [109]. EMT enables epithelial cells to acquire migratory capacity by downregulating epithelial markers and cell-cell junctions while upregulating mesenchymal markers [112]. Tumor cells undergoing mesenchymal migration exhibit an elongated, spindle-shaped morphology with pseudopodial and filopodial extensions. Mesenchymal movement is characterized by extended protrusions and a polarized cell body (Table 2). While leading-edge protrusions advance rapidly, rear detachment is often delayed, resulting in slower net translocation velocity [113]. This mode of migration relies on cytoskeletal contractility, integrin-mediated adhesion to the ECM, and proteolytic matrix degradation. Focal adhesion kinase (FAK) and Src family kinases regulate cytoskeletal reorganization and the formation of focal adhesions [114]. Leading-edge protrusions are Rac-dependent, and cell movement is achieved through coordinated formation and turnover of integrin-mediated adhesions [115].

**Table 2:** Comparative overview of mesenchymal and ameboid cell migration.

	Mesenchymal Migration	Ameboid Migration
**Morphology**	Fusiform, elongated	Rounded, highly deformable
**Motility mode**	Anchorage through adhesion complexes and integrins	Penetration of spaces through cortical actomyosin contraction
**Matrix degradation**	Protease-dependent	Protease-independent
**Migration rate**	Low	High
**Regulatory pathway**	Rac1	Rho/ROCK

Cells may also migrate in an amoeboid-like mode. Amoeboid migration is characterized by rapid, crawling-like movement in which the cell adopts a flexible, dynamic shape (Table 2). Unlike mesenchymal migration, it does not rely on strong focal adhesions or extensive extracellular matrix degradation. Instead, it is driven by rapid cytoskeletal rearrangements (actin-myosin contractions) and cytoplasmic flow, allowing cells to squeeze through narrow ECM gaps.

Three distinct ameboid motile variants have been described: (i) highly dynamic cells with thin protrusions and no blebs that migrate at high speed; (ii) slower cells with a blebbing morphology; and (iii) proteolytic cells featuring short protrusions and lower motility [113]. Amoeboid motility based on blebbing manifests in two forms. The first is polarized migration—also known as leader or stable bleb-based motility—in which cells migrate directionally toward a dominant bleb. These cells achieve higher migration speeds and maintain a stable, polarized, balloon-like morphology driven by cortical contractility. The second form is non-polarized motility, which results in erratic and chaotic cellular movements [116].

Cellular plasticity enables transitions between mesenchymal and amoeboid states (mesenchymal-amoeboid transition, MAT, and amoeboid-mesenchymal transition, AMT), processes regulated by protease activity, cell-matrix interactions, and Rho/Rac-mediated actomyosin dynamics. In addition, AMT and MAT, alongside EMT and mesenchymal-epithelial transition (MET), drive reversible shifts between collective and single-cell migration. These collective-individual transitions reshape cell-cell adhesions, cell-matrix interactions, cytoskeletal architecture, and pericellular proteolysis [107]. Both biochemical composition and biophysical properties of the ECM strongly influence migratory behaviour [117,118,119]. A recent study using a synthetic stromal matrix showed that environmental stiffness and fiber density act as independent cues governing cell movement and triggering transitions between migratory states, including single-cell amoeboid/mesenchymal motility, cluster migration, and linear strand formation. The availability of adhesive ligands promotes a partial mesenchymal shift, enabling collective invasion as multicellular strands that evade apoptotic death. In contrast, in the absence of such fibers and adhesive ligands, cells preferentially adopt an individual, amoeboid-like mode of migration [117].

Tumor metastasis and therapeutic resistance are strongly promoted by MAT. Because cancer cells can readily switch between mesenchymal and amoeboid phenotypes, they can dynamically adapt their invasion strategies. Targeting the signaling pathways underlying MAT may therefore represent a promising strategy to inhibit metastatic dissemination [120].

Furthermore, collective cell migration involves groups of cells that retain cell–cell junctions and move together in a coordinated manner [121]. This process relies on actin cytoskeleton dynamics, integrin-mediated cell-ECM, and pericellular proteolysis, which remodels the ECM [122]. Cadherins, immunoglobulin superfamily members, and gap junctions stabilize cell–cell contacts [123]. During collective invasion, heterogeneous tumor cell clusters polarize into leading and trailing regions. Leader cells, positioned at the invasive front, exhibit distinct gene expression profiles, morphology, and enhanced proliferative, invasive, and metastatic potential compared with follower cells [124]. They direct migration through Rac-driven protrusive activity and integrin-dependent ECM adhesion and overexpress key proteases such as MMP-14 and cathepsin B [107,125].

Cancer cells typically do not maintain a single mode of migration but instead adapt their migratory behaviour to environmental challenges by switching between collective and single-cell dissemination modes [126]. The ability of cancer cells to switch between different motility strategies in response to contextual cues, including those induced by therapies targeting cancer metastasis, has been proposed as a potential mechanism underlying resistance to pharmacological approaches [107].

## 5 Cytoskeletal Dynamics: Structural Components and Molecular Pathways

Cell migration is regulated by a complex network of signaling pathways that includes lipid second messengers, small GTPases, kinases, cytoskeleton-modifying proteins, and motor proteins. To sustain persistent movement, cells establish a front (leading edge) and a rear (trailing edge), with distinct signaling pathways driving membrane protrusion at the front and retraction at the rear. In most cases, cell orientation is biased by external gradients of soluble factors and adhesive ligands, which induce an asymmetric distribution of intracellular signaling events. The actin cytoskeleton plays a major role in generating protrusive structures and transmitting the forces required for cell translocation.

In cells that predominantly migrate in a mesenchymal manner, a key step in the migratory cycle is the formation of stable anchoring sites known as focal adhesions. These structures transmit adhesive forces between the cytoskeleton and the extracellular matrix through transmembrane adhesion receptors. Focal adhesions have two crucial properties: they locally activate signal transduction pathways, thereby reinforcing polarity during directional migration, and they respond dynamically to tensile stress, reflecting the mechanical forces generated both within and outside the cell and the mechanical properties of the cell and the extracellular matrix [127].

### 5.1 Actin-Binding Proteins

ABPs are central regulators of actin filament polymerization and depolymerization. They organize filaments into higher-order structures and control complex filament dynamics. Through these activities, ABPs enable the actin cytoskeleton to rapidly respond to intracellular and extracellular signals, supporting essential cellular processes such as cell shape and motility, muscle contraction, intracellular trafficking, and pathogenesis [128].

Actin nucleators initiate the formation of new actin filaments through a process called nucleation. Three major classes of nucleators have been identified: the Arp2/3 complex, formins, and Spire. The Arp2/3 complex is involved in actin branching and is composed of seven subunits; two of these, ARP2 and ARP3, structurally mimic actin monomers and serve as a template for the initiation of branched, Y-shaped filament networks [129].

Although traditionally described as a unitary entity, the Arp2/3 complex is now recognized as a family of eight iso-complexes composed of distinct isoforms of ARP3, ARPC1, and ARPC5, with unique biochemical properties and selective roles in lamellipodia, invadopodia, and membrane trafficking [130]. This isoformic heterogeneity introduces a level of functional specificity that is only beginning to be explored in the current literature, particularly regarding how upstream signals govern iso-complex composition in tumor cells and how this variability influences matrix degradation versus general cell motility.

The Arp2/3 complex requires activation by nucleation-promoting factors (NPFs), including WASP, WAVE/SCAR, and cortactin [131]. However, the functional hierarchy and redundancy among these NPFs in cancer invasion remain poorly defined, as they are recruited by distinct upstream signals, including Cdc42, Rac1, and phosphatidylinositol 4,5-bisphosphate (PtdIns(4,5)P_2_), and operate within distinct subcellular compartments [132]. Dysregulation of the Arp2/3 complex has been linked to metastasis, and its components are frequently upregulated in tumors, where they are associated with cell invasion and metastatic behavior [132]. However, this role is not universal. Loss of Arp2/3 activity has been shown to induce DNA damage, chromosomal segregation defects, micronucleus formation, and cellular senescence through p53 activation and p21-mediated G1 arrest [133]. This apparent paradox, whereby Arp2/3 overexpression promotes invasion while its loss triggers genomic instability, highlights the context-dependent nature of Arp2/3 function in tumor biology and underscores that its role is more complex than a simple pro-metastatic effect, representing a significant unresolved controversy in the field.

Regulators of G-actin polymerization include proteins such as profilin and thymosin β4, whereas cofilin promotes F-actin depolymerization. Cofilin is a small, ubiquitous actin-binding protein that interacts with both G- and F-actin, increasing the number of free barbed ends available for polymerization while accelerating depolymerization. Through these activities, cofilin promotes the formation of lamellipodia, invadopodia, and filopodia involved in cancer cell migration and invasion [134]. It also enhances the nucleation activity of Arp2/3 complex [135]. LIMK1 and LIMK2 regulate cofilin by phosphorylating it at Ser3, thereby inhibiting its actin-binding activity [136].

Beyond this canonical role, cofilin has been implicated in vasculogenic mimicry in breast cancer cells [137], adding a pro-angiogenic dimension to its established pro-invasive functions. Furthermore, emerging evidence links cofilin-1 in mitochondrial dynamics and apoptosis regulation under oxidative stress conditions, suggesting that its overall impact on tumor biology may vary substantially depending on the cellular microenvironmental context [138]. This complexity is not yet fully integrated into current mechanistic models. LIMK1 has also recently been identified as a direct kinase of β-catenin, acting synergistically with CDK5 to phosphorylate β-catenin at Ser191 and promote its nuclear translocation, thereby activating metastatic signaling pathways independently of cofilin [139]. This non-canonical function suggests that LIMK1 may function as a multi-substrate oncogenic node and raises the possibility that its contribution to tumor progression has been systematically underestimated in studies focused primarily on the LIMK–cofilin axis.

An additional regulatory layer, largely overlooked in the cancer biology literature, involves the phosphatase Slingshot (SSH), which dephosphorylates and reactivates cofilin. Loss of SSH function results in accumulation of phosphorylated cofilin and F-actin, phenocopying LIMK overactivation, and indicating that the dynamic balance between LIMK and SSH, rather than LIMK activity alone, is a critical determinant of cofilin-driven motility [136]. However, the role of SSH across different tumor types and its potential as a therapeutic target remains insufficiently explored. Expression profiling studies have identified the cofilin pathway as a major determinant of metastasis, with several genes upregulated during metastatic progression acting either within this pathway or as downstream effectors [140].

Capping and severing proteins regulate actin filament dynamics by preventing the addition or loss of G-actin monomers at F-actin filaments. Examples include CapZ and gelsolin [141]. The group of bundling and cross-linking proteins includes, among others, fascin (FSCN), a major actin-bundling protein. FSCN1 is virtually absent in normal epithelial tissues but is dramatically upregulated in human carcinomas, where it crosslinks actin filaments into parallel bundles and localizes to filopodia, invadopodia, and podosomes, correlating with increased cell motility and invasiveness [142]. Despite extensive documentation of FSCN1 overexpression across tumor types, the molecular mechanisms driving its upregulation during malignant transformation, including potential roles for super-enhancers, m6A RNA modification, or RNA-binding proteins, remain poorly understood and constitute an underexplored area of research [142]. Another unresolved issue concerns the functional interplay between FSCN1 and tropomyosin: isoforms encoded by TPM2 have recently been shown to directly regulate fascin-1 bundling activity [143], suggesting that the balance between pro-invasive FSCN1 and metastasis-suppressive TPMs is mechanistically coupled. The molecular basis of this reciprocal regulation, and whether it can be exploited as a vulnerability in invasive cancer cells, remains to be established.

Myosins are a superfamily of actin-dependent molecular motor proteins that convert energy derived from ATP hydrolysis into mechanical force through interactions with microfilaments. They play essential roles in multiple aspects of eukaryotic cell motility. Myosins consist of three functional domains: an N-terminal head domain responsible for actin binding and ATP hydrolysis; a neck domain that interacts with calmodulin and light chains; and a class-specific C-terminal tail domain involved in cargo transport along actin filaments, signal transduction, and membrane interactions [144].

Proteins that anchor actin filaments to the plasma membrane include talin, kindlin, ezrin, radixin, and moesin, among others. Talin is a major component of the focal adhesion complex, linking β-integrin cytoplasmic tails to F-actin [145], and has been implicated in focal adhesion dynamics, cell migration and invasion, and metastasis through activation of the Src-FAK complex [146].

F-actin stabilizing and regulatory proteins, such as tropomyosins (TPMs), are best known for their role in calcium-dependent contraction in muscle cells; however, in non-muscle cells, they primarily maintain cytoskeletal stability [147]. Mammals express four TPM genes that generate nearly 40 isoforms through alternative splicing [148]. All isoforms form coiled-coil α-helical dimers that assemble into continuous cables along both sides of the actin filament helix [149], acting as gatekeepers that regulate the access of other ABPs to microfilaments [150]. TPM expression profiles are profoundly altered in malignancy [151]. However, an important unresolved question is the extent to which isoform composition differentially shapes the behavior of distinct tumor cell populations. It also remains unclear whether specific TPM isoforms may serve as independent prognostic biomarkers or selective therapeutic vulnerabilities, particularly in the context of their antagonistic interplay with FSCN1 [143].

### 5.2 Rho GTPases: Regulators of the Cytoskeleton in Cell Migration

The Rho family of small GTPases and their effectors constitute a central regulatory network that controls actin cytoskeleton organization and dynamics, thereby regulating cell motility and contributing to metastatic progression [115]. Rho GTPases belong to the Ras superfamily and are grouped into three major subfamilies: Rho, Rac, and Cdc42. Like other Ras-related proteins, they function as molecular switches that cycle between an active GTP-bound state and an inactive GDP-bound state. Upstream signals promote GDP release and GTP loading, inducing conformational changes in the effector-binding region that modulate interactions with downstream targets according to nucleotide-bound status and subcellular localization [152].

Three classes of molecules tightly regulate Rho GTPase activity: guanine nucleotide exchange factors (GEFs), which activate Rho GTPases by catalyzing GDP–GTP exchange; GTPase-activating proteins (GAPs), which inactivate them by stimulating intrinsic GTP hydrolysis; and guanine nucleotide dissociation inhibitors (GDIs), which bind inactive Rho GTPases, sequestering them in the cytosol and preventing signaling. GDIs also protect Rho proteins from ubiquitination and degradation [152]. The frequent overexpression or hyperactivation of Rho GTPases in cancer, despite the rarity of direct mutations in solid tumors, indicates that their dysregulation primarily arises from altered control by GEFs, GAPs, and GDIs [152]. Among GEFs, ECT2, PREX1, VAV1, and TIAM1 have been implicated in tumor growth and progression across multiple cancer types through hyperactivation of Rho GTPase signaling [153]. Conversely, several RhoGAPs function as tumor suppressors. DLC1, the best-characterized RhoGAP in cancer, is frequently downregulated through copy number loss, transcriptional silencing, or proteasomal degradation mediated by the ubiquitin ligase HECTD1, leading to aberrant RhoA activation and invasive behavior [154]. Importantly, however, some RhoGAPs can also act as oncogenes in specific tumor subtypes, as demonstrated for ARHGAP11A and RACGAP1 in basal-like breast cancer [155], highlighting the context-dependent functions of GAP proteins in cancer. The specific GEF–GTPase and GAP–GTPase interaction networks dysregulated in each tumor type, as well as the mechanisms by which individual GEFs confer signaling specificity despite activating the same GTPase, remain incompletely characterized and represent a major mechanistic gap in the field [155].

Once activated, Rho GTPases interact with a wide array of effector molecules. Among these, the best characterized is ROCK, a serine/threonine kinase that drives cytoskeletal remodeling. The ROCK family comprises two isoforms: ROCK1 and ROCK2, which share high sequence identity but have partially non-redundant roles. Upon activation, RhoA-GTP binds the C-terminal region of ROCK, inducing stress-fiber formation, focal adhesion assembly, cell-cell junction maturation, and cell-cycle regulation [156]. ROCK enhances myosin contractility by phosphorylating myosin light chain (MLC) and myosin phosphatase target subunit 1 (MYPT1), promoting actomyosin-based force generation necessary for cell migration [157].

Despite sharing most known substrates, ROCK1 and ROCK2 are functionally distinct: ROCK1 preferentially promotes actomyosin contractility through MLC2 phosphorylation, whereas ROCK2 more strongly regulates actin stabilization through cofilin signaling [158]. Importantly, studies using isoform-specific silencing in non-small cell lung cancer have shown that although inhibition of either isoform alone suppresses anchorage-independent growth, suppression of cofilin phosphorylation requires loss of both isoforms, suggesting functional overlap for specific substrates but non-overlapping roles in tumor growth [159]. This distinction has direct consequences for the interpretation of data generated with pan-ROCK inhibitors, which cannot discriminate between isoform-specific contributions.

Other key Rho effectors include LIMK, which phosphorylates cofilin and prevents actin filament disassembly [160], as well as ERM proteins, CRMP2, calponin, and adducin [161]. Additional regulators such as Tau and MAP2 contribute to microtubule structure and dynamics [162]. Finally, RhoA-driven stress fiber formation sequesters angiomotin (AMOT), an inhibitor of YAP, thereby promoting YAP nuclear translocation and activation of YAP/TAZ signaling [163], adding a mechanotransduction dimension to RhoA oncogenic activity that extends beyond cytoskeletal remodeling.

Despite sharing 85% sequence identity, the three members of the Rho subfamily (RhoA, RhoB, RhoC) have distinct and sometimes opposing cellular functions [164,165]. Aberrant activation or overexpression of RhoA and RhoC has been reported in several cancers, including urinary tract tumors [166], cervical cancer [167], and gastric cancer [168]. However, the oncogenic role of RhoA is not universal: genome sequencing studies have identified recurrent loss-of-function RhoA mutations, most notably G17V, in angioimmunoblastic T-cell lymphoma, adult T-cell leukemia/lymphoma, and diffuse large B-cell lymphoma, where RhoA appears to function as a tumor-suppressor rather than an oncogene [169,170]. This context dependency fundamentally challenges the view of RhoA as a uniformly pro-tumorigenic GTPase.

RhoB presents an even more complex picture: although it exerts tumor-suppressive effects in several solid tumors [165], its unique C-terminal post-translational modifications determine subcellular localization to endosomes, multivesicular bodies, and the nucleus. These compartments not shared by RhoA or RhoC, and underlie context-dependent oncogenic and tumor-suppressive functions, earning RhoB the designation of an “oncojanus” gene [165,171]. The molecular determinants that shift RhoB from tumor suppressive to oncogenic functions in specific cellular contexts remain incompletely understood. In contrast, atypical Rho GTPases beyond the canonical RhoA/Rac1/Cdc42 triad remain comparatively understudied in cancer, despite emerging evidence implicating them in proliferation, apoptosis, vesicular trafficking, and tumor progression [172].

**Figure 2:**
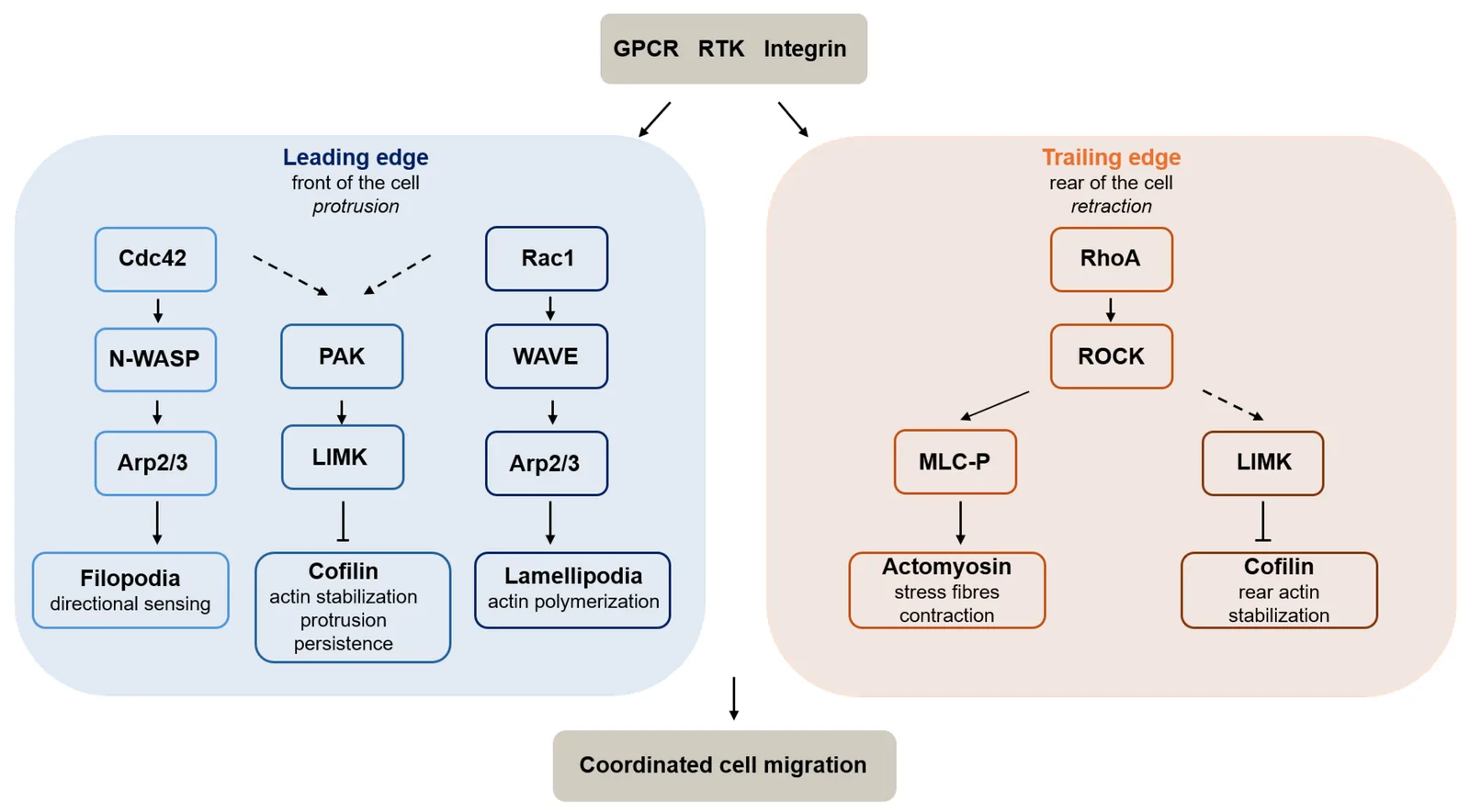
Regulation of actin cytoskeleton dynamics in a migrating cell by Rho GTPases and their downstream effectors. At the leading edge, Cdc42 activates N-WASP, and Rac1 activates the WAVE complex, both converging on the Arp2/3 complex to promote branched actin nucleation and the formation of filopodia and lamellipodia, respectively. Cdc42 and Rac1 also activate PAK kinases, which phosphorylate LIMK, resulting in cofilin inhibition and actin filament stabilization. At the trailing edge, RhoA signals through ROCK to phosphorylate and activate LIMK, which inhibits cofilin and stabilizes actin filaments, while also phosphorylating MLC to enhance actomyosin contractility and promote rear retraction.

Rac reorganizes the actin cytoskeleton to form broad lamellipodia that drive cell motility, whereas Cdc42 promotes the formation of actin-rich protrusions and filopodia that sense chemotactic cues and guide directional movement. Rac activates the WAVE complex, triggering Arp2/3-dependent actin polymerization, while Cdc42 activates WASP, which in turn interacts with the Arp2/3 complex to induce filopodia formation. Both Rac and Cdc42 activate PAK kinases, which stimulate actin assembly through LIMK and regulate myosin phosphorylation and contractility via pathways involving MLCK and other myosin components. They also interact with IQGAP to influence cell–cell adhesion and activate PI3K, whose lipid products enhance Rac GEF activity in a positive feedback loop that promotes migration. Additionally, Cdc42 activates MRCKs, which function alongside Rho kinases to increase myosin phosphorylation [173,174]. Rac and Cdc42 are also overexpressed in a variety of malignancies [175,176,177]. A critical limitation of current mechanistic knowledge in this area is that most functional studies on Rho GTPases in cancer have been conducted in two-dimensional cell culture systems or xenograft models, which fail to recapitulate the three-dimensional architecture, biomechanical properties, and stromal interactions of the tumor microenvironment [178]. Integrating Rho GTPase signaling studies into more physiologically relevant platforms, such as patient-derived organoids and tumor-on-chip systems, represents an important priority for the field. These models may better capture signaling redundancy and pathway plasticity within Rho GTPase networks, which enable tumor cells to adapt to inhibition of individual signaling nodes and are difficult to recapitulate in simplified experimental systems [172]. Fig. 2 summarizes the regulation of actin cytoskeleton dynamics in migrating cells by Rho GTPases and their downstream effectors.

### 5.3 Integrins and Focal Adhesions: Key Connectors between the ECM and the Cytoskeleton

Integrins constitute a large family of cell-surface transmembrane heterodimeric receptors that regulate numerous biological functions. This family comprises 18 α and 8 β subunits, which assemble into 24 distinct integrin heterodimers. Integrins are frequently overexpressed and perform diverse functions across cancer types. For example, β1 integrin pairs with 12 different α subunits to form a diverse set of heterodimers that mediate some of the most critical functions within the integrin family [179].

Integrins play a key role in supporting all hallmarks of cancer, including sustained proliferative signaling, evasion of growth suppression, enabling replicative immortality, resistance to cell death, induction of angiogenesis, activation of invasion and metastasis, deregulation of cellular metabolism, and evasion of immune destruction [180]. Integrins and integrin-dependent pathways participate in nearly every stage of cancer progression.

Metastasis typically begins when cancer cells break the basement membrane, a process supported by integrin-driven upregulation and activation of matrix-degrading proteases. Integrins also enable stromal invasion and migration, aided by cancer-associated fibroblasts (CAFs) that remodel the ECM and guide tumor cell movement by generating pro-migratory tracks, depositing fibronectin, aligning fibronectin fibers, or physically pulling cancer cells out of the primary tumor.

Circulating tumor cells (CTCs) undergo anchorage-independent growth and exhibit anoikis resistance through pathways that involve altered integrin signaling. For metastasis to succeed, CTCs must adhere to the vasculature of distant organs and extravasate into the perivascular tissue, a process often facilitated by thrombus-mediated fibronectin recruitment and integrin activation. After extravasation, integrins interact with ECM components at the secondary site, ultimately determining whether disseminated cells will proliferate or remain dormant [180].

An emerging and mechanistically important dimension of integrin biology concerns mechanosensing: ECM stiffness, which increases progressively during tumor progression due to collagen crosslinking and CAF-driven fibrosis, is sensed primarily through integrin clustering and downstream activation of FAK and RhoA–ROCK signaling, promoting focal adhesion maturation, PI3K activity, and nuclear YAP/TAZ translocation. This mechanotransduction axis represents a positive feedback loop in which tumor-driven ECM stiffening further amplifies integrin signaling and invasive behavior. However, the exact signaling pathways by which integrins activate YAP/TAZ in cancer cells remain incompletely understood and somewhat controversial [180]. Furthermore, single-molecule studies have recently shown that distinct integrin subtypes require markedly different tension thresholds to support cell spreading and drive qualitatively different cytoskeletal remodeling programs [181], suggesting that integrin subtype composition in tumor cells may determine not only adhesion strength but also the mode of force transmission and downstream signaling. The implications of this subtype-specific mechanobiology for tumor invasion and dormancy reactivation have not yet been systematically investigated.

Focal adhesions (FAs) are specialized, dynamic structures through which cells physically connect their cytoskeleton to the extracellular matrix. These sites consist of integrin clusters and an extensive network of structural and signaling proteins that link integrins to actomyosin filaments. The interplay between actomyosin contractile forces and FA dynamics establishes a balance between adhesion and contraction, which strongly influences migration velocity [182].

FAK is one of the most extensively studied regulators of FAs. This non-receptor tyrosine kinase acts as a central integrator, sensing multiple extracellular signals and coordinating numerous downstream signaling pathways. Its integrative function is demonstrated by its activation in response to a variety of extracellular stimuli, including integrins, growth factor receptors, G-protein-coupled receptors, and cytokine receptors [183]. FAK is involved in the assembly and turnover of FAs through the recruitment of paxillin and talin, actin remodeling, the expression and activation of MMPs, and the regulation of anoikis [184]. These functions are consistent with the frequent overexpression of FAK in various types of cancer and its association with poor prognosis [185].

Beyond its well-established cytoplasmic kinase role, FAK translocates to the nucleus where it exerts both kinase-dependent and kinase-independent scaffolding functions that are mechanistically distinct from its adhesion-related activities [186]. In a kinase-independent manner, nuclear FAK binds p53, GATA4, and Runx1 to regulate gene expression involved in cell cycle progression, inflammation, and survival; notably, nuclear FAK promotes p53 degradation via MDM2-mediated ubiquitination, contributing to tumor cell growth [187]. More recently, FAK has been shown to control chromatin accessibility genome-wide at promoter and enhancer regions, including the IL-33 locus, which regulates anti-tumor immunity, a function that depends on FAK kinase activity [188]. These distinct nuclear roles raise the question of whether current ATP-competitive inhibitors, which block kinase activity but leave scaffolding functions intact, are sufficient to fully suppress nuclear FAK signaling [183].

### 5.4 The Src Pathway in Cytoskeletal Dynamics

Src is a member of a family of non-receptor membrane-associated tyrosine kinases. It catalyzes the transfer of the terminal phosphate group from ATP to specific tyrosine residues on protein substrates, thereby transmitting signals from the extracellular environment to intracellular biochemical pathways. These pathways can either activate nuclear factors, leading to transcriptional responses, or target cytoplasmic components, resulting in cytoskeletal reorganization. When oncogenically activated, Src can stimulate increased cell growth and survival, contributing to tumor formation, while also promoting actin cytoskeleton reorganization and reducing cell-cell and cell-matrix adhesion, thereby facilitating cell motility and invasion [189]. During cancer progression, Src activity is frequently elevated, yet activating mutations or gene amplification are uncommon in human tumors. Instead, dysregulated upstream control of Src, including reduced activity of its negative regulator C-terminal Src kinase (CSK), and aberrant activation of tyrosine phosphatases such as PTPN11/SHP2, which dephosphorylate the inhibitory Tyr530 residue, releases Src from its autoinhibited conformation and is thought to play a major role in its aberrant activation in cancer [190]. An important and underappreciated regulatory dimension is provided by the Src N-terminal regulatory element (SNRE), an intrinsically disordered region unique to each Src family member, whose non-canonical regulatory functions remain poorly understood. The SNRE can modulate Src activity and substrate selectivity independently of the canonical SH2/SH3 regulatory domains, and its tumor cell-specific roles suggest that broad kinase inhibition may fail to recapitulate the selectivity required for effective cancer targeting [191]. Furthermore, beyond its canonical signaling functions, Src has been implicated in metabolic reprogramming, regulation of the inflammatory response within the tumor microenvironment, and acquisition of chemotherapy resistance [191].

Beyond its role as a signaling molecule, FAK serves as a scaffold that recruits Src and its substrates to sites of integrin engagement. Clustering of integrins following cell-matrix contact recruits FAK to nascent adhesion sites via FAT domain interactions with paxillin and talin, leading to conformational activation and autophosphorylation at Y397. Tyrosine phosphorylation of FAK at Y397 creates a high-affinity binding site for the Src SH2 domain, leading to Src recruitment, activation, and formation of a stable FAK–Src complex. Within this complex, Src phosphorylates FAK at Y576, Y577, Y861, and Y925, fully activating the kinase and generating docking sites for downstream effectors including CAS, paxillin, and p190RhoGAP, which are essential for actin cytoskeletal reorganization and cell migration [187,192]. At the leading edge of migrating cells, the complex coordinates adhesion assembly and recruits CAS, which, via Crk and DOCK180, activates Rac. Rac drives peripheral actin polymerization to generate protrusive forces and can also activate JNK, increasing MMP-2 and MMP-9 expression to promote pericellular proteolysis and invasion [193,194]. Paxillin can recruit a trimeric signaling complex at adhesion sites, comprising Rac and the Cdc42 effector protein PAK, which mediates ERK/MAPK activation and regulates the actin cytoskeleton by phosphorylating MLC and MLCK. At the rear of migrating cells, the FAK–Src complex recruits ERK/MAPK and calpain, thereby promoting adhesion disassembly and enhancing motility and invasion [194]. Src also suppresses Rho activity at the leading edge via phosphorylation of p190RhoGAP, reducing contractility and facilitating protrusion, while Rho activity predominates at the rear to drive actomyosin contraction. This spatial regulation of Rac and Rho downstream of integrin–Src signaling ensures coordinated cell migration [195].

**Figure 3:**
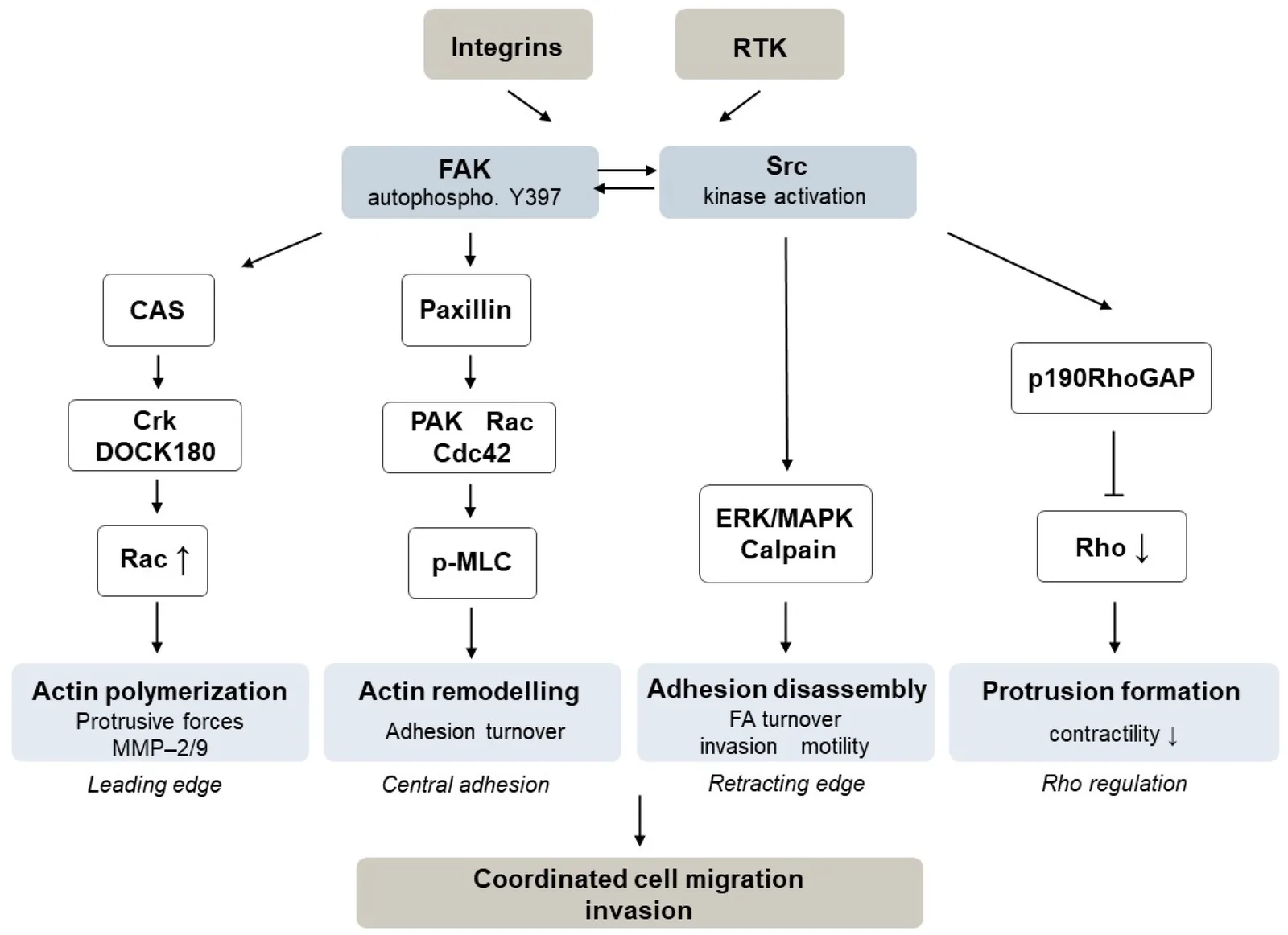
Role of the FAK-Src complex in coordinated cell migration and invasion. Following integrin and RTK engagement, FAK undergoes autophosphorylation at Y397 and recruits Src. The resulting FAK-Src complex signals through four parallel branches: (1) at the leading edge, FAK activates CAS, which signals via Crk/DOCK180 to promote Rac1 activation, actin polymerization, protrusive force generation, and MMP-2/9-dependent matrix degradation; (2) the FAK-Src complex cooperates with paxillin to activate PAK, Rac, and Cdc42, driving actin remodeling and focal adhesion turnover at central adhesions; (3) at the retracting edge, Src activates ERK/MAPK signaling and calpain, promoting adhesion disassembly, FA turnover, motility, and invasion; (4) Src phosphorylates p190RhoGAP, suppressing RhoA activity, reducing contractility, and facilitating protrusion formation. Together, these pathways converge to coordinate directional cell migration and invasion.

It should be noted, however, that the spatial Rac/Rho gradient model described above is largely based on two-dimensional *in vitro* systems. In three-dimensional and *in vivo* contexts, tumor cells frequently switch between mesenchymal and amoeboid migration modes, the latter driven by high Rho/ROCK activity throughout the cell rather than being restricted to the rear [100,108], challenging the universality of this model and underscoring a significant gap between the mechanistic framework established in simplified systems and the complexity of invasion in native tumor microenvironments. A critical unresolved question concerns how FAK–Src signaling integrates with mechanosensory inputs from the ECM: FAK activation is enhanced on stiff matrices through a mechanosensitive loop involving integrin clustering, actomyosin tension, and FAK–Src co-activation. Notably, the temporal relationship between tensional changes and FAK activation is context dependent. FAK activation can precede tension changes at nascent adhesions, whereas in mature adhesions, applied tension precedes full FAK activation [183]. Finally, while FAK’s scaffolding functions at focal adhesions are well established, its kinase-independent nuclear activities in transcriptional regulation and immune evasion represent an expanding area of biology that is mechanistically distinct from cytoplasmic signaling and may contribute to tumor progression independently of integrin engagement (Fig. 3) [187].

## 6 Cancer Plasticity: The Role of Tumor Microenvironment

The bidirectional communication between the tumor microenvironment (TME) and the cancer cell cytoskeleton represents a fundamental regulatory axis that governs cancer plasticity and metastatic cascade [5,196]. Beyond its structural complexity, the TME acts as a functional hub where biochemical signals, such as cytokines, chemokines, growth factors and proteases, and physical stressors, such as acidity and hypoxia, converge to drive malignancy [197]. The heterogeneity of the TME, influenced by the tumor’s anatomical site and stage, directly impacts cancer cell responses to pharmacological treatments [198]. Central to this process is continuous crosstalk between tumor cells and the associated immune landscape, which engages specific signal transduction pathways to evade host defenses. In addition, nutrient deprivation and impaired blood flow within neoplastic lesions paradoxically create a protective milieu for tumor development and dissemination.

The reciprocal relationship between TME and cancer cells functions as a continuous feedback loop: the TME provides biochemical signals and mechanical cues, such as ECM stiffness and interstitial fluid pressure, which are sensed and transduced by the cytoskeleton via focal adhesions and mechanosensory complexes. In response, the cytoskeleton undergoes rapid structural remodeling, enabling cells to transition between different states of plasticity. These cytoskeletal rearrangements do not merely facilitate passive adaptation; they actively contribute to remodeling of the TME through the secretion of matrix-degrading enzymes and the deposition of new ECM components. This reciprocity ultimately establishes a self-sustaining cycle that promotes cellular motility, intravasation, and the survival of circulating tumor cells, thereby laying the groundwork for metastatic colonization [199]. Cytoskeletal remodeling within the TME depends on a highly integrated regulatory network that extends beyond the structural components of actin filaments, microtubules, and intermediate filaments. Rather, it emerges from the coordinated activity of signaling mediators, ion channels, membrane proteins, adhesion molecules, and mechanotransduction pathways, which collectively enable cancer cells to dynamically adapt their morphology and migratory behavior in response to environmental cues [200,201,202,203,204]. The following examples illustrate how distinct features of the TME regulate this interconnected network to promote cancer cell plasticity and metastatic progression.

The physical properties of the ECM act as a powerful regulator of the cancer cell cytoskeleton. ECM stiffness is a key determinant of tumor cell invasion and migration [205]. Increased ECM rigidity promotes an invasive phenotype and accelerates tumor progression through multiple signaling pathways, making cells more aggressive. Cancer cells sense ECM stiffness through transmembrane receptors. In stiff matrices (as commonly observed in breast and pancreatic cancer), integrins cluster into focal adhesions. This clustering triggers the activation of Rho-family GTPases, which in turn promotes assembly of contractile actin stress fibers. Increased cytoskeletal tension allows cells to exert greater force on the ECM, facilitating their ability to “plow” through dense tissue [206,207]. The migratory phenotype of cancer cells is often dictated by the balance of Rho GTPases activity: Rac activity primarily drives mesenchymal migration, whereas increased RhoA activity promotes transition to amoeboid movement [208,209]. Additionally, increased matrix stiffness supports the formation of a detyrosinated microtubule network, which is functionally important for the localization of RNAs required for cell movement [207].

Tumor cores are frequently hypoxic, a condition that forces cells to adapt their structural framework to survive and disseminate. Hypoxia induces expression of specific tubulin isoforms, most notably βIII-tubulin [210]. This isoform increases microtubule dynamic instability, promoting cancer progression by regulating invadopodia formation and migration, while also contributing to resistance to Taxanes [211,212,213,214]. Low oxygen levels can also upregulate MAPs such as Tau or stathmin, which remodel microtubule tracks to favor rapid cytoskeletal reorganization, enabling directional migration toward nutrient-rich blood vessels [215,216].

TME is typically acidic due to high lactate production. Changes in intracellular and extracellular pH affect the activity of several pH sensors that collectively regulate different aspects of cell migration. These include guanine nucleotide exchange factors for the small GTPase Cdc42 involved in cell polarity [217], talin-actin interactions [218], FAK-dependent adhesion dynamics [219], as well as gelsolin and cofilin-mediated actin polymerization [220]. These mechanisms regulate actin cytoskeletal turnover, determining whether a cell remains stationary or adopts a highly motile state [221].

Moreover, EMT-driven plasticity is influenced by soluble factors released by neighboring stromal cells, including fibroblasts, immune cells, and endothelial cells, which reprogram the cytoskeleton through EMT signaling. For example, under the influence of TGF-β or HGF, cells downregulate keratins, key components of epithelial structural integrity, and upregulate vimentin. Vimentin provides the mechanical flexibility required for survival during intravasation into the bloodstream. In addition, the microenvironment promotes actin cytoskeletal reorganization into invadopodia, facilitating extracellular matrix degradation and invasion.

## 7 Antimetastatic Therapeutic Approaches Targeting the Cytoskeleton

As reported, MTAs induce mitotic arrest or apoptosis; however, they can also inhibit the metastatic process through specialized molecular mechanisms that are frequently active at sub-toxic concentrations.

The migrastatic actions of MTAs exerted at low, non-toxic doses are linked to several mechanisms including:

- Disruption of FA Turnover: Proper microtubule dynamics are indispensable for regulating the assembly and disassembly cycle of FAs. MTAs stabilize these structures, thereby preventing the cell from detaching and advancing [222]. Low doses of these agents reduce the number of peripheral microtubules, compromising leading-edge stability and the repositioning of the microtubule-organizing center (MTOC) toward the direction of movement [223,224,225];
- Loss of directionality (polarity destabilization): without a flexible, remodeling microtubule network, cells lose their structural compass. As a result, they cannot maintain a designated leading edge, which prevents them from executing directional steering [225,226];
- Inhibition of invadopodia and metalloproteinase trafficking: to invade healthy tissues, cancer cells form membrane protrusions known as invadopodia. MTAs prevent microtubule recruitment required for their elongation into functional structures [227]. In addition, microtubules act as tracks for the transport and exocytosis of MMPs, such as MMP-2 and MMP-9; MTAs disrupt this trafficking, preventing the secretion of proteolytic enzymes required for basement membrane degradation [228];
- Interference with oncogenic signaling pathways: MTAs act as migration inhibitors by interfering with the delivery of signaling molecules that drive invasive phenotypes. For example, the Hedgehog (Hh) signaling pathway, which promotes EMT, depends on microtubule integrity for the localization of its components to the primary cilium; MTAs suppress EMT by disrupting this signaling hub [229];
- Inhibition of HIF-1α trafficking and anti-angiogenesis: at low, non-cytotoxic concentrations, MTAs block the migration of endothelial cells. This occurs through the disruption of dynein-mediated transport of the HIF-1α factor to the nucleus, subsequently reducing VEGF production [230]. Without VEGF, the primary stimulus for the focal adhesion turnover necessary for both tumor migration and the formation of new blood vessels (neo-angiogenesis) is lost [231].

While MTAs, particularly taxanes, are highly effective at halting mesenchymal migration, continuous exposure to these drugs induces cancer cells to undergo a rapid behavioral switch termed MAT. Instead of utilizing the slow, proteolysis-dependent (anchor-and-chew) mesenchymal mode, cells morph into a rounded, highly flexible phenotype. Amoeboid migration relies predominantly on the actin cytoskeleton and the Rho/ROCK signaling pathway [232], bypassing the need for microtubules. Cells utilize actomyosin contractions to effectively squeeze, slide, and slither through gaps in the ECM, without requiring integrin-mediated attachment or enzymatic tissue digestion [233]. Because amoeboid migration does not rely on stable microtubule tracks, cells utilizing this mode are largely resistant to the migrastatic effects of taxanes, allowing them to bypass the taxane blockade and continue metastasizing [234].

Similar to taxanes, Vinca alkaloids are potent tools in cancer treatment. However, they disrupt the microtubule network from the opposite structural direction. When applied at low, non-toxic, migrastatic doses, Vinca alkaloids are exceptionally effective at sabotaging mesenchymal migration, yet they trigger the exact same compensatory escape mechanism in cancer cells. While taxanes act by over-stabilizing and freezing microtubule tracks, MDAs, as Vinca derivatives, bind directly to tubulin dimers at a specific interface, preventing their assembly into polymers [235]. At higher doses, they completely unravel the microtubule network into paracrystalline aggregates. At low, sub-toxic migrastatic doses, they drastically suppress the microtubule growth rate, essentially dismantling the cellular transport infrastructure [226]. Because mesenchymal migration is a slow, methodical process that relies entirely on an intact, polarizing structural skeleton, stripping a cell of its microtubules via MDAs fundamentally impairs its motility by inducing a loss of protrusion support, halting cargo delivery, and disrupting cell polarity [236]. Nevertheless, as seen for MTAs, these drugs ultimately promote the switch to amoeboid squeezing.

Consequently, it is hypothesized that a “double-lock” migrastatic strategy could successfully prevent metastasis by neutralizing the behavioral plasticity of cancer cells. Specifically, while MTAs or MDAs are deployed to abrogate mesenchymal migration, focal adhesion turnover, and directionality, the concurrent administration of Rho/ROCK or actin inhibitors would block the alternative amoeboid switch [101]. By simultaneously targeting both the microtubule-driven mesenchymal machinery and the actin-driven amoeboid machinery, this combined approach is expected to effectively strip cancer cells of their migratory adaptability.

### 7.1 Innovative Antimetastatic Compounds

Since tumor invasion and metastasis convert a localized primary lesion into a severe, systemic, and potentially lethal disease, therapeutically targeting or preventing this cascade could significantly improve patient outcomes [237]. Unlike normal cells, metastatic tumor cells lack contact inhibition, enabling them to breach otherwise impermeable biological barriers. Consequently, targeting cancer cell motility represents a promising strategy to reduce systemic dissemination and potentially minimize the need for aggressive, high-toxicity treatments such as conventional chemotherapy.

Molecules belonging to this class interfere with invasion and migration, as well as with the mechanisms underlying the metastatic potential of cancer cells. Unlike conventional cytostatic drugs, which primarily act by blocking cell cycle progression and proliferation, these compounds modulate cytoskeletal dynamics and cell–matrix interactions, thereby directly impacting the ability of tumor cells to migrate, invade surrounding tissues and colonize distant sites [238]. In Table 3 we summarize the cytoskeleton-targeting compounds with anti-metastatic properties.

**Table 3:** Cytoskeleton-targeting compounds with anti-metastatic properties. Clinical trial registration data retrieved from ClinicalTrials.gov (https://clinicaltrials.gov/); FDA approval data retrieved from the FDA Drug Database (https://www.accessdata.fda.gov/scripts/cder/daf/).

Drugs	Class	Target	Mechanism of Action	Development Stage
NP-G2-044	Small molecule fascin inhibitor	Actin-binding proteins	Fascin inhibitor	Clinical trial
Jasplakinolide	Marine-derived cyclodepsipeptide	F-actin	Actin stabilization	Preclinical studies
Chondramide	Myxobacterial macrolide	F-actin	Actin stabilization; PKCε inactivation-mediated cancer cell-selective apoptosis	
Cytochalsins	Fungal metabolites	Actin filament barbed ends	Actin destabilization	
Latrunculin	Marine sponge macrolides	G-actin monomers		
Blebbistatin	Myosin II inhibitor	Non-muscle myosin II (NMIIA, NMIIB)	ATPase activity inhibitor	Preclinical studies
TR100	Tropomyosin inhibitor	Tm5NM1, Tm5NM2	Tropomyosin-actin filament destabilization	Preclinical studies
Withaferin A	Withanolides	Intermediate filaments	Vimentin inhibitor	Preclinical studies
Y-27632	Selective ROCK inhibitors	ROCK1, ROCK2	ATP-competitive inhibitor	Preclinical studies
Fasudil	ROCK inhibitors			Clinically approved (Anticancer use under evaluation in preclinical studies)
CCT129253				Preclinical studies
AT13148	Multi AGC kinase inhibitors	ROCK1, ROCK2, AKT, PKA, p70S6K		Clinical trial (Discontinued due to narrow therapeutic index and unfavorable pharmacokinetic properties)
Pyr1	LIMK inhibitors	LIMK1, LIMK2	ATP-competitive inhibitor	Preclinical studies
IN10018	Kinase domain inhibitors	FAK	ATP-competitive molecule	Clinical trial
Defactinib		FAK, PYK2		
GSK2256098		FAK		
Conteltinib		FAK, PYK2, ALK		
APG2449		FAK, ALK, ROS1		
Dasatinib	Multi target tyrosine kinase inhibitor	BRC-ABL, SFK, KIT	ATP-competitive molecule	Clinically approved
Bosutinib		BRC-ABL, SFK, c-KIT, PDGFR		
Ponatinib		BRC-ABL, Src, KIT, RET, FLT3, PDGFR, FGFR		
Saracatinib		c-Src, ABL		Clinical trial
Tirbanibulin	Dual Src/tubulin inhibitor	Src, Tubulin	Non-ATP-competitive molecule	Clinically approved (Anticancer use clinical trial)

Migrating cancer cells undergo profound and continuous morphological changes that depend on dynamic remodeling of the actin cytoskeleton [238]. This process involves the formation of actin-based protrusive structures at the leading edge, such as lamellipodia and filopodia, the establishment of new adhesions to the extracellular matrix, and the generation of contractile forces required for rear retraction and forward translocation of the cell body [239]. Throughout these steps, actin filaments are continuously assembled and disassembled in a tightly regulated manner. In addition, actin contributes to the formation of specialized invasive structures such as invadopodia and podosomes, which are actin-rich, adhesive and proteolytically active domains formed at the cell–ECM interface by mesenchymally migrating cells [240]. In concert with myosin motors, actin filaments generate contractile forces that drive cell motility. Actin and myosin assemble into higher-order structures, such as stress fibers and bleb-like protrusions, which are essential for efficient migration. A central regulatory event in actomyosin contractility is activation of the RhoA, which in turn activates ROCK [232].

Focal adhesion–associated proteins such as Src and FAK play pivotal roles in coordinating adhesion dynamics, cytoskeletal remodeling and extracellular matrix-derived signaling. By integrating mechanical and biochemical cues, these kinases regulate the assembly and disassembly of adhesion sites, the organization of actin structures and the migratory behavior of tumor cells [223,224].

**Figure 4:**
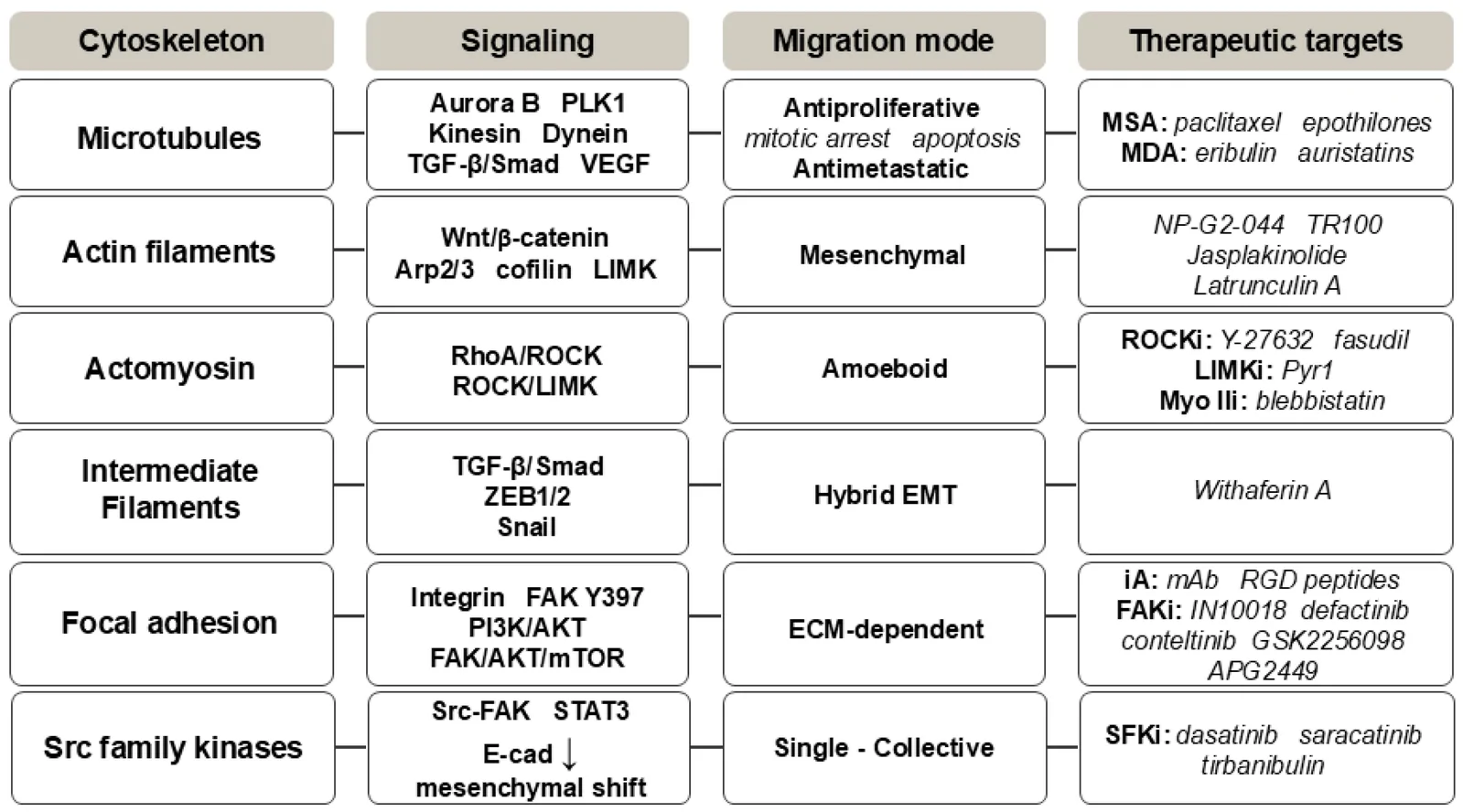
Summary of the major cytoskeletal components involved in cancer cell migration, their associated signaling pathways, the migration modes they regulate, and representative therapeutic targets. Six systems are covered: microtubules, actin filaments, actomyosin, intermediate filaments, focal adhesions, and Src family kinases.

Therefore, compounds that target cytoskeleton organization, actomyosin contractility, or key regulators such as Rho/ROCK, Src and FAK have the potential to specifically impair cancer cell motility and invasiveness [238] (Table 3). Such agents may complement traditional antiproliferative therapies by limiting metastatic dissemination, which remains a major cause of cancer-related mortality [241]. Fig. 4 summarizes the major cytoskeletal components involved in cancer cell migration, their associated signaling pathways, the migration modes they regulate, and representative therapeutic targets.

### 7.2 Microfilament-Targeting Agents

#### 7.2.1 Modulating the Function of Actin-Binding Proteins

Numerous ABPs, both directly and indirectly, have been shown to significantly influence the migratory and metastatic phenotypes of tumor cells.

The actin-bundling protein FSCN is a key structural component of filopodia, cellular sensory organelles that interface with the extracellular microenvironment and contribute to fundamental processes such as cell adhesion, spreading and migration in three-dimensional environments [242,243,244]. Owing to this central role in regulating cell motility and invasion, FSCN has emerged as a promising molecular target for cancer therapy. The FSCN inhibitor NP-G2-044 blocks its actin-binding and actin-bundling activities, thereby inhibiting breast cancer cell migration and suppressing metastasis in mouse models [245]. In non-small cell lung cancer, NP-G2-044 has also been shown to reduce metastatic dissemination and enhance the efficacy of cisplatin and PD-1 inhibitors, at least in part through inhibition of the Wnt/β-catenin signaling pathway [246]. This compound is currently under clinical investigation in patients with advanced or metastatic solid tumors, both as monotherapy and in combination regimens (NCT07109414, NCT05023486).

#### 7.2.2 Modulating Actin Cytoskeleton Polymerization

Actin filaments have attracted considerable interest as potential targets for antimetastatic therapies because they play a fundamental role in cancer cell motility and metastasis-associated invasiveness. Actin-targeting compounds interfere with actin dynamics either by stabilizing filaments, thereby promoting actin polymerization, or by inhibiting filament assembly, resulting in filament destabilization. However, because actin is an essential cytoskeletal component of normal cells, these agents often exhibit poor specificity and may induce severe adverse effects, including cardiotoxicity, nephrotoxicity, and hepatotoxicity. Consequently, the development of actin-targeting drugs has largely remained at the preclinical stage, and to date no such agents have received FDA approval.

Jasplakinolide is a marine sponge–derived compound that binds F-actin, stabilizing filaments and promoting actin polymerization. It has demonstrated antitumor activity in models of Lewis lung carcinoma, where it reduces metastatic dissemination, as well as in prostate carcinoma models [247].

Chondramide, another actin-stabilizing agent produced by *Chondromyces crocatus*, has been shown to inhibit breast carcinoma cell invasion and suppress lung metastasis dissemination [248,249]. Its antitumor and antimetastatic effects are mediated through disruption of actin-dependent cytoskeletal remodeling and induction of cancer cell–selective apoptosis via PKCε inactivation [250].

Cytochalasins are fungal metabolites that bind to and cap the barbed ends of actin filaments, thereby preventing further polymerization. More than 60 cytochalasins have been identified; among these, cytochalasins B and D predominantly exert cytostatic rather than cytotoxic, effects in a variety of cancer cell lines [251].

Latrunculins are marine sponge–derived compounds that inhibit actin polymerization by sequestering G-actin monomers. Latrunculin A is the most potent member of this class and impairs actin assembly by forming a 1:1 complex with G-actin while also inhibiting nucleotide exchange on the monomer. *In vitro* viability assays have demonstrated dose-dependent anticancer effects of latrunculin A, and *in vivo* studies have shown reduced peritoneal dissemination of human gastric cancer cells in mouse models [252].

#### 7.2.3 Modulating Actin Cytoskeleton Contractility

Blebbistatin is a small-molecule inhibitor that selectively targets the ATPase activity of non-muscle myosin II [253,254], thereby impairing actomyosin contractility and the generation of forces required for cell migration and invasion. By disrupting myosin II–driven cytoskeletal dynamics, blebbistatin reduces cancer cell motility, extracellular matrix invasion, and formation of invasive structures [255]. In various tumor models, pharmacological inhibition of non-muscle myosin II by blebbistatin has been shown to attenuate metastatic potential and alter tumor cell mechanics, supporting the concept of myosin II as a promising target in antimetastatic therapy [256,257].

TR100 is an anti-tropomyosin compound that selectively targets the Tm5NM1 and Tm5NM2 tropomyosin isoforms, which are overexpressed in several cancer types. Tropomyosin is associated with actin filaments and stabilizes them by preventing depolymerization. TR100 selectively destabilizes actin filaments in malignant cells by disrupting the interaction between Tm5NM1/2 and actin, thereby reducing cancer cell motility and survival. *In vivo* studies have further demonstrated that TR100 suppresses melanoma and neuroblastoma growth without inducing hepatotoxicity or cardiotoxicity, which are common adverse effects associated with current actin-targeting agents. These findings highlight the potential of TR100 as a relatively low-toxicity actin-targeting compound capable of limiting tumor metastasis [5].

It is important to emphasize that most therapies targeting actin dynamics are still at the preclinical stage.

### 7.3 Intermediate Filament-Targeting Agents

Oncogenic transformation alters the IF composition of cells, leading to changes in the expression of specific IF proteins. Among these, vimentin is frequently overexpressed in several cancers, including prostate, gastrointestinal, and breast tumors. In solid tumors, vimentin promotes metastatic progression by participating in the cytoskeletal reorganization associated with EMT and by regulating pro-EMT signaling pathways. Vimentin has also been used as a marker of pre-metastatic cells undergoing EMT; accordingly, high vimentin expression is associated with poor prognosis in patients with solid tumors [258]. In addition, vimentin contributes to angiogenesis and modulation of vascular immune checkpoints when secreted by tumor endothelial cells [259,260]. Although the role of vimentin in cancer is not yet fully understood, its consistent overexpression in aggressive disease [261], its involvement in multiple metastasis-related processes [262,263], and its dispensability for normal adult survival make it an attractive therapeutic target.

Withaferin A (WA), a naturally occurring steroidal lactone isolated from *Withania somnifera*, exhibits anti-angiogenic and anticancer activities, in part through inhibition of vimentin function [264]. WA directly interacts with vimentin by targeting the Cys328 residue, leading to aggregation of vimentin filaments. In combination with F-actin, this interaction disrupts the vimentin cytoskeleton, resulting in changes in cell shape, decreased motility, and increased phosphorylation of vimentin at Ser38 [265]. WA has demonstrated antitumor activity in both *in vitro* and *in vivo* studies. It inhibits tumor growth by inducing G_2_/M cell cycle arrest and promoting apoptotic cell death through downregulation of Bcl-2 proteins. In addition, WA suppresses angiogenesis by targeting VEGFR signaling [266] and inhibits tumor migration by stopping EMT. In the MDA-MB-231 xenograft model and in MMTV-neu transgenic mice, WA exhibited anti-metastatic activity by inhibiting vimentin expression [266]. Furthermore, WA has been shown to sensitize cancer cells to chemotherapy and radiotherapy. Combination treatment with other anticancer agents has frequently produced enhanced therapeutic effects. For example, co-treatment with sorafenib synergistically suppressed thyroid cancer growth, potentiated G_2_/M arrest, and enhanced apoptosis through caspase-3 activation and PARP cleavage [267,268]. Moreover, WA co-administration reduced the required dose of chemotherapeutic agents, thereby lowering toxicity to normal cells, as demonstrated with cisplatin. In ovarian cancer cell lines, the IC_50_ of cisplatin decreased from 40 μM to 12 μM when combined with 1.5 μM WA. Despite its promising anticancer properties, WA remains under preclinical investigation because additional pharmacokinetic, safety, and toxicological studies are still required [267].

Similar to therapies targeting actin dynamics, therapeutic strategies directed against intermediate filaments remain largely confined to the preclinical stage of development.

### 7.4 Rho/ROCK Pathway-Targeting Agents

As direct inhibition of actin polymerization is associated with high toxicity in normal tissues, recent studies have focused on indirect strategies targeting upstream regulators of actin dynamics. Among these approaches, inhibition of ROCK and LIMK has emerged as a promising strategy for selectively impairing the metastatic potential of cancer cells. ROCK is a key regulator of the actomyosin cytoskeleton and controls cellular force generation, invasion, proliferation, and contractility. Numerous studies have demonstrated that ROCK inhibition suppresses tumor progression and metastasis in both *in vitro* systems and *in vivo* models of multiple solid cancers [269].

Y-27632 is a pyridine derivative that competitively inhibits ATP binding to ROCK while also affecting other kinases. It was the first ROCK inhibitor to be identified. Y-27632 has been shown to significantly suppress the growth and migration of oral squamous cell carcinoma (OSCC) cells and to induce autophagy through modulation of the AKT/mTOR signaling pathway [270]. It has also been shown to modulate key mechanotransduction pathways in endometrial cancer (EC). In EC tissues, extracellular matrix stiffness is significantly increased compared with normal endometrium and correlates with elevated ROCK1 expression. ROCK1 promotes YAP1 phosphorylation, nuclear localization and transcriptional activity, thereby driving aggressive tumor phenotypes characterized by enhanced proliferation, migration, invasion, and reduced apoptosis. Pharmacological inhibition of ROCK1 with Y-27632 reverses these pro-tumorigenic effects by suppressing tumor growth, inducing cell-cycle arrest, and restoring apoptosis. Moreover, Y-27632 enhances sensitivity to chemotherapy and radiotherapy and promotes macrophage-mediated phagocytosis, thereby strengthening antitumor immune responses. In hormone-resistant EC models, Y-27632 restores responsiveness to progesterone, and xenograft studies have demonstrated superior antitumor activity when combined with progesterone compared with monotherapy [271]. Y-27632 has also been found to inhibit the ROCK–MRCK signaling in bladder cancer, thereby reducing actomyosin-mediated contractility and suppressing cancer cell proliferation and invasion [272].

Fasudil, currently the only ROCK inhibitor clinically approved for cerebrovascular disease in Japan and China, has demonstrated antitumor activity in several cancer models. In small cell lung cancer (SCLC), fasudil decreases the proportion of cells in the S phase while increasing accumulation in the G_0_/G_1_ and G_2_/M phases, accompanied by reduced expression of c-Myc and cyclin D1. It also promotes tumor maturation, as indicated by increased sensitivity to starvation-induced apoptosis. *In vivo* further studies demonstrated significant inhibition of tumor growth [273]. In ovarian cancer models, fasudil suppresses tumor cell motility and invasion by inhibiting ROCK activation and disrupting cytoskeletal remodeling, including lysophosphatidic acid–induced formation of stress fibers and focal adhesions. In ovarian cancer xenograft models, fasudil treatment significantly reduced intraperitoneal tumor burden [274].

CCT129253 and AT13148, two selective ROCK inhibitors, have also been shown to negatively regulate melanoma cell invasion, while CCT129253 additionally inhibits metastatic dissemination *in vivo*. Among these compounds, AT13148 is the only ROCK inhibitor that has progressed into clinical trials for advanced solid tumors (NCT01585701); however, clinical development has been limited by a narrow therapeutic index and unfavorable pharmacokinetic properties [275].

LIMKs regulate the actin cytoskeleton architecture through phosphorylation and inactivation of actin-depolymerizing factors belonging to the ADF/cofilin family. In addition, LIMKs regulate microtubule dynamics independently of actin filament turnover, although the molecular basis of this activity remains unknown. Inhibition of LIMKs results in microtubule stabilization together with severing and disorganization of actin microfilaments. Given the central roles of both actin and microtubule cytoskeletons in cell division and motility, pharmacological inhibition of LIMKs is expected to exert anti-metastatic effects, and LIMKs are increasingly recognized as promising therapeutic targets in cancer.

4-pyridocarbazolone (Pyr1) is a highly selective LIMK inhibitor that stabilizes microtubules and induces cell-cycle arrest during the S and G_2_/M phases. By inhibiting cofilin phosphorylation, Pyr1 also blocks actin microfilament dynamics. Pyr1 is active *in vitro* in both paclitaxel-sensitive and paclitaxel-resistant cancer cell lines and displays therapeutic efficacy *in vivo* in a murine L1210 leukemia model, while being well tolerated [276].

Overall, because ROCK and LIMK inhibitors disrupt key regulators of actin remodeling and cytoskeletal plasticity, targeting these upstream signaling pathways may represent an effective and selective strategy for inhibiting metastatic dissemination.

### 7.5 Integrin-Src-FAK Axis-Targeting Agents

#### 7.5.1 Blocking Integrin Functions

Although integrins are not structural components of the cytoskeleton, they play a central role in its organization by linking extracellular signals to the dynamics of actin and microtubules. Based on their role in promoting cancer cell invasion, metastasis, and tumor angiogenesis, integrin antagonists have been explored as indirect cytoskeleton-targeting agents in cancer therapy. Recent findings suggest that integrins also mediate or regulate additional functions relevant to cancer cells, including dormancy, metabolism, survival, therapy resistance, EMT, fibrosis, cancer stemness, exosome homing, and pre-metastatic niche formation [248]. Monoclonal antibodies and synthetic RDG peptides, which block integrin function by occupying the ligand-binding site, have been used in clinical trials. Preclinical studies have shown that targeting αvβ3, αvβ5, and β1 integrins can prevent tumor angiogenesis, reduce tumor growth, and limit metastatic spread. Despite promising *in vitro* and *in vivo* preclinical studies indicating that integrins can be effectively targeted, either alone or in combination with radio-, chemo-, or immune therapies, clinical outcomes to date are not encouraging [277,278].

#### 7.5.2 Inhibiting Focal Adhesion Kinase

FAK inhibitors can be divided into three main categories: (1) kinase domain inhibitors, (2) allosteric inhibitors, and (3) proteolysis-targeting chimeras (PROTACs). All compounds currently under investigation in clinical trials belong to the kinase domain inhibitor class. These agents are ATP-competitive molecules that bind to FAK and compete with ATP, thereby inhibiting FAK signaling and downstream pathway activation [187,279].

IN10018 (ifebemtinib, BI853520) is a selective FAK inhibitor that shows enhanced activity in tumors with a mesenchymal phenotype, particularly those characterized by elevated E-cadherin expression. Preclinical studies demonstrated that IN10018 rapidly and persistently suppresses FAK autophosphorylation in tumor tissue and inhibits cancer cell growth *in vitro* and *in vivo*, including in breast cancer, malignant pleural mesothelioma, and ovarian cancer [280,281]. IN10018 reduces tumor burden, cell proliferation, and tumor microvascular growth, and can inhibit EMT via modulation of the FAK/AKT/mTOR pathway [281,282]. In murine models of pancreatic cancer, combination therapy with IN10018 and radiotherapy enhances the therapeutic response and promotes immune cell infiltration, including CD8^+^ T cells and macrophages [283]. IN10018 is being evaluated in several clinical trials, including two completed Phase I studies (NCT01905111, NCT01335269), which reported manageable safety, favorable pharmacokinetics, and anti-tumor activity in patients with advanced or metastatic solid malignancies, including modest antitumor activity at a maximum tolerated dose of 200 mg once daily in selected non-hematologic cancers [284,285].

Defactinib (VS-6063) is an efficient, reversible dual inhibitor of FAK and Proline-rich tyrosine kinase 2 (PYK2). It suppresses FAK autophosphorylation at Tyr397 in a time- and dose-dependent manner. Across multiple tumor models characterized by FAK overexpression, defactinib has been shown to inhibit tumor cell growth and survival primarily via blockade of PI3K/AKT and downstream signaling pathways [286,287]. In prostate cancer, the combination of defactinib with docetaxel significantly reduces the viability of docetaxel-resistant cells *in vitro* and *in vivo*, consistent with the observed positive association between FAK expression and advanced tumor stage in primary human prostate cancers [288]. In non-small cell lung cancer, defactinib synergizes with the EGFR inhibitor osimertinib to more effectively inhibit AKT activation and induce apoptosis, suggesting a promising strategy to overcome EGFR-TKI resistance [289]. Similarly, in pancreatic ductal adenocarcinoma (PDAC) with FAK overexpression, defactinib exhibits anti-proliferative and anti-migratory effects and acts synergistically with paclitaxel to inhibit tumor cell proliferation both *in vitro* and *in vivo* [290]. In uterine serous carcinoma (USC), reactive oxygen species (ROS) drive FAK phosphorylation and promote invasion and metastasis via the ROS–FAK–PAX axis. In patient-derived orthotopic xenograft models of USC, defactinib markedly suppresses tumor growth [291]. Defactinib is under investigation in multiple Phase I, II, and III clinical trials. Among them, study NCT0787033 demonstrated an acceptable safety profile, with adverse events that were mild to moderate and reversible in patients with advanced solid malignancies [292]. Moreover, in the study NCT01951690, defactinib monotherapy showed modest clinical activity in heavily pretreated patients with KRAS-mutant NSCLC [293].

GSK2256098 is an orally bioavailable inhibitor with high selectivity for FAK. It effectively suppresses CPNE8-mediated FAK signaling, thereby impairing gastric cancer cell migration and metastasis in preclinical models [294], and inhibits the proliferation of multiple PDAC cell lines [295]. The antitumor efficacy of GSK2256098 appears to be associated with aberrant expression of specific genes or proteins, as patients with PTEN-mutant endometrial cancer exhibit markedly improved responses compared with those harboring PTEN wild-type tumors [296]. This compound is under evaluation in phase I and II clinical trials. Study NCT01138033 demonstrated that GSK2256098 is tolerable in patients with relapsed glioblastoma. The compound was detected at low concentrations in normal brain tissue but at substantially higher levels within tumor tissue, consistent with blood brain-barrier disruption in the tumor microenvironment [297]. A Phase Ib study (NCT01938443) demonstrated that co-administration of GSK2256098 with trametinib, a MEK inhibitor, increases trametinib exposure while maintaining an acceptable safety profile [298]. Given the high prevalence of NF2 mutations in meningiomas and the synthetic lethal interaction between FAK inhibition and NF2 loss, the Phase II study NCT01523014 evaluated the efficacy of GSK2256098 in a genomically selected cohort of patients with recurrent or progressive meningiomas. GSK2256098 was well tolerated and achieved an improved 6-month progression-free survival rate in patients with NF2-mutated tumors compared with that in the control group [299].

Conteltinib is a multi-kinase inhibitor targeting FAK, PYK2, and anaplastic lymphoma kinase (ALK). Phase I clinical data in patients with advanced ALK-positive NSCLC demonstrated manageable safety, favorable pharmacokinetics, and preliminary antitumor activity (NCT02695550) [300]. Preclinical studies indicate broad anticancer effects primarily mediated by FAK pathway inhibition, including suppression of tumor growth and lung metastasis in breast cancer models [301], as well as inhibition of FAK signaling in liver and lung cancers. In liver cancer, conteltinib inhibits tumor growth by blocking hypoxia-activated IGF1R–YAP signaling [302] and overcomes sorafenib resistance via YAP pathway suppression [303].

APG-2449 is a novel multikinase inhibitor with activity against oncogenic alterations of ALK, ROS proto-oncogene 1 receptor tyrosine kinase (ROS1), and FAK. In esophageal squamous cell carcinoma (ESCC) models, the combination of APG-2449 and ibrutinib suppresses tumor cell survival and invasion, induces G_1_/S cell-cycle arrest, and promotes apoptosis, effects that are mechanistically linked to reduced phosphorylation of the MEK/ERK and AKT signaling pathways [304]. In ovarian xenografts models, APG-2449 sensitizes tumors to paclitaxel by reducing CD44^+^ and ALDH1^+^ cancer stem cell populations, including in carboplatin-insensitive tumors. In EGFR-mutant NSCLC xenografts, APG-2449 enhances EGFR TKI–mediated tumor inhibition and, when combined with osimertinib and the MEK inhibitor trametinib, overcomes resistance to Osimertinib [305]. This compound is currently under investigation in Phase I clinical trials. Study NCT03917043 demonstrated favorable preliminary safety, pharmacokinetic, and efficacy profiles in patients with TKI-untreated or second-generation ALK inhibitor–resistant NSCLC. Notably, higher baseline levels of tumor phosphorylated FAK were associated with greater clinical benefit [306].

#### 7.5.3 Modulating Src Family Kinase

Dasatinib is an oral tyrosine kinase inhibitor (TKI) approved for the treatment of chronic myeloid leukaemia (CML) in the chronic, accelerated, and blast phases, as well as Philadelphia chromosome–positive (Ph+) acute lymphoblastic leukaemia (ALL) in adults and children. It received initial approval from the FDA and EMA in 2006. Like other *BCR-ABL*–targeting TKIs (imatinib, nilotinib, bosutinib, and ponatinib), dasatinib inhibits the Breakpoint Cluster Region-Abelson (BCR-ABL) oncogene, a driver of leukaemogenesis [307]. BCR-ABL is an aberrant fusion protein generated by a reciprocal chromosomal translocation, resulting in the *BCR-ABL* fusion gene. In addition, dasatinib inhibits KIT and members of the Src family kinases (SFKs). It was initially approved as an alternative to existing therapies, including imatinib (the first marketed BCR-ABL TKI, introduced in 2001), in patients with intolerance or resistance to prior treatments. It has been reported to be approximately 325-folds more potent than imatinib [308].

Bosutinib, another second-generation *BRC-ABL1* TKI, was approved by the FDA in 2012 and by the EMA in 2013. In addition to *BRC-ABL1*, it targets SFKs, platelet-derived growth factor (PDGF) receptors, and c-KIT. The third-generation *BRC-ABL1* TKI, ponatinib, received FDA and EMA approval in 2012 and 2013, respectively [309]. Ponatinib also targets RET, Fms-like tyrosine kinase 3 (FLT3), KIT, Src, and members of the fibroblast growth factor receptor (FGFR) and PDGFR families [310]. Both bosutinib and ponatinib are approved for patients with chronic, accelerated, or blast phase Ph+ CML who are resistant or intolerant to prior therapy, including imatinib. The availability of *BCR-ABL1* TKIs has progressively improved the prognosis of patients with CLM, bringing life expectancy closer to normal levels [309].

Saracatinib (AZD0530) is an orally available, highly selective dual c-Src/Abl kinase inhibitor. It binds to the ATP-binding site and is classified as a type II inhibitor, stabilizing the inactive conformation of the kinase. The compound is currently under investigation in Phase I–III clinical trials in patients with advanced or metastatic solid tumors, both as monotherapy and in combination with chemotherapeutic agents [311].

Tirbanibulin (KX2-391) is a first-in-class inhibitor of Src kinase signaling and tubulin polymerization. It received FDA approval in 2020 for the topical treatment of actinic keratosis on the face or scalp. Tirbanibulin has also been investigated in early-phase clinical trials for various cancers, including acute myeloid leukemia, prostate cancer, and other solid tumors, due to the critical role of its targets in cancer progression [312].

## 8 Conclusion and Future Perspective

The cytoskeleton plays a central role in the metastatic cascade, acting as a dynamic scaffold that determines cell shape and motility and regulates numerous associated cellular processes. Tumor cell migration depends on four tightly coordinated events: protrusion, adhesion, contraction, and retraction, each critically controlled by cytoskeletal components and their associated regulatory networks. Actin drives membrane protrusion through lamellipodia, filopodia, and invadopodia, which are essential for cell motility and extracellular matrix degradation during invasion; together with myosin, actin also generates contractile forces. The other two major cytoskeletal filament systems, microtubules and intermediate filaments, further contribute to metastatic behavior by regulating mitosis, cytoskeletal remodeling through crosstalk with actin, and cell morphology and mechanical properties. Within this network, Rho family GTPases such as Cdc42 and Rac, together with signaling molecules including Src and FAK, are key regulators of protrusion formation, while Src and FAK, in cooperation with integrins, orchestrate adhesion site assembly and turnover. RhoA and its downstream effectors primarily control actomyosin-mediated contraction.

Given the central role of both structural cytoskeletal components and their regulatory networks in metastatic progression, significant efforts have focused on developing agents that selectively target these molecules and, ideally, tumor-specific alterations that characterize malignant cells. However, the clinical translation of many cytoskeleton-targeting compounds has been limited by significant toxicity to normal cells, the emergence of drug resistance, and the remarkable adaptive capacity of cancer cells. To date, among cytoskeleton-directed agents, only microtubule-targeting compounds have gained clinical approval for the treatment of various tumors.

Future cytoskeleton-targeted cancer therapies are shifting toward greater precision and adaptability while minimizing systemic toxicity, with an emphasis on selective regulators, metastasis-specific mechanisms, combinatorial strategies, and advanced delivery systems. Rather than directly targeting core cytoskeletal filaments, current approaches focus on more selective and pharmacologically accessible regulators, including actin-binding proteins (such as Arp2/3 and tropomyosins), microtubule-associated proteins (MAPs), and intermediate filament components such as vimentin. There is also growing interest in co-targeting signaling pathways linked to cytoskeletal dynamics to enhance therapeutic efficacy and overcome resistance to conventional microtubule-targeting agents.

Conventional cancer therapies face significant hurdles due to drug resistance, metastatic dissemination, immune evasion, enhanced invasive and migratory capacities, and colonization of secondary sites. To counteract resistance, combination or synergistic therapeutic strategies offer a more promising approach by simultaneously disrupting multiple oncogenic pathways. Accordingly, an optimal therapeutic regimen should enhance anti-tumor immune responses, inhibit cancer cell migration from the primary tumor, and suppress invasion, seeding, and subsequent proliferation.

Combination approaches that target multiple pathways at lower doses, integrating cytoskeletal drugs with kinase inhibitors, DNA-damaging agents, or immunotherapies, are being explored to reduce toxicity and limit resistance. In parallel, advanced drug delivery systems, including antibody–drug conjugates, liposomes, nanoparticles, and other tumor-targeted platforms, are emerging as key tools to improve specificity, stability, and safety. Nanomaterials capable of selectively disrupting the tumor cytoskeleton while sparing normal tissues represent a particularly promising strategy, offering dual effects on tumor proliferation and metastatic potential. A central future direction is the use of cytoskeletal profiling as a source of biomarkers for prognosis and patient stratification, enabling more personalized therapeutic strategies.

## Data Availability

Not applicable.

## Abbreviations

ABP	Actin-binding protein
ADC	Antibody–drug conjugate
ALK	Anaplastic lymphoma kinase
ALL	Acute lymphoblastic leukaemia
AMOT	Angiomotin
BCR-ABL	Breakpoint Cluster Region-Abelson oncogene
CA-4	Combretastatin A4
CA-4P	Combretastatin A4 phosphate
CAF	Cancer-associated fibroblast
CBS	Colchicine-binding site
CML	Chronic myeloid leukaemia
CTC	Circulating tumor cell
DM1	Synthetic derivative of maytansine
EC	Endometrial cancer
ECM	Extracellular matrix
ESCC	Esophageal squamous cell carcinoma
EMT	Epithelial-mesenchymal transition
ERM	Ezrin-radixin-moesin
FA	Focal adhesion
FAK	Focal adhesion kinase
FGFR	Fibroblast growth factor receptor
FH	Formin homology domain
FLT3	Fms-like tyrosine kinase 3
FSCN	Fascin
GAP	GTPase-activating protein
GDI	Guanine nucleotide dissociation inhibitor
GEF	Guanine nucleotide exchange factor
IF	Intermediate filament
IQGAP	IQ motif containing GTPase-activating protein
LIMK	LIM kinase
MAP	Microtubule-associated protein
MAT	Mesenchymal-amoeboid transition
MDA	Microtubule-destabilizing agent
MDR	Multidrug resistance
MET	Mesenchymal-epithelial transition
MF	Microfilament
MLC	Myosin light chain
MLCK	Myosin light chain kinase
MMAE	Monomethyl auristatin E
MMP	Metalloproteinase
MSA	Microtubule-stabilizing agent
MT	Microtubule
MTA	Microtubule-targeting agent
MTOC	Microtubule-organizing center
MYPT1	Myosin phosphatase 1
NMII	Non-muscle myosin II
NPF	Nucleation-promoting factor
NSCLC	Non–small-cell lung cancer
OSCC	Oral squamous cell carcinoma
PDA	Pancreatic ductal adenocarcinoma
PDGF	Platelet-derived growth factor
Pgp	P-glycoprotein
Ph	Philadelphia chromosome
PROTAC	Proteolysis-targeting chimeras
PYK2	Proline-rich tyrosine kinase 2
Pyr1	4-pyridocarbazolone
ROCK	Rho-associated coiled-coil-containing kinase
ROS1	ROS proto-oncogene 1 receptor tyrosine kinase
RTK	Receptor tyrosine kinase
sALCL	Systemic anaplastic large cell lymphoma
SCAR	Suppressor of cyclic AMP-receptor
SCLC	Small cell lung cancer
SFK	SRC family kinase
SSH	Slingshot phosphatase
TKI	Tyrosine kinase inhibitor
TME	Tumor microenvironment
TPM	Tropomyosin
USC	Uterine serous carcinoma
VDA	Vascular disrupting agent
WA	Withaferin A
WASP	Wiskott–Aldrich syndrome protein
WAVE	WASP-family verprolin-homologous protein
